# Nanosecond mid-infrared pulse generation via modulated thermal emissivity

**DOI:** 10.1038/s41377-019-0158-6

**Published:** 2019-06-05

**Authors:** Yuzhe Xiao, Nicholas A. Charipar, Jad Salman, Alberto Piqué, Mikhail A. Kats

**Affiliations:** 1 Department of Electrical and Computer Engineering, University of Wisconsin–Madison, Madison, WI 53706 USA; 2 Naval Research Laboratory, 4555 Overlook Ave. SW, Washington, DC 20375 USA; 3 Department of Materials Science and Engineering, University of Wisconsin–Madison, Madison, WI 53706 USA; 4 Department of Physics, University of Wisconsin–Madison, Madison, WI 53706 USA

**Keywords:** Ultrafast photonics, Mid-infrared photonics

## Abstract

We demonstrate the generation of nanosecond mid-infrared pulses via fast modulation of thermal emissivity enabled by the absorption of visible pump pulses in unpatterned silicon and gallium arsenide. The free-carrier dynamics in these materials result in nanosecond-scale modulation of thermal emissivity, which leads to nanosecond pulsed thermal emission. To our knowledge, the nanosecond thermal-emissivity modulation in this work is three orders of magnitude faster than what has been previously demonstrated. We also indirectly observed subnanosecond thermal pulses from hot carriers in semiconductors. The experiments are well described by our multiphysics model. Our method of converting visible pulses into the mid infrared using modulated emissivity obeys different scaling laws and can have significant wavelength tunability compared to approaches based on conventional nonlinearities.

## Optics: A pulsating development in infrared technologies

American scientists have created ultrafast pulses of infrared radiation from the surface of two semiconducting materials for use in a range of new applications in optical communications, spectroscopy, and sensing. Fast pulses of light in the visible and near-infrared range have seen use in a variety of applications, including telecommunications and materials processing. However, generating pulses in the mid-infrared range has proved challenging as existing techniques require complex and expensive equipment that produce low-power pulses with limited tunability. Now, Yuzhe Xiao, Mikhail Kats, and colleagues from the University of Wisconsin–Madison and the Naval Research Laboratory in the United States have produced ultrafast mid-infrared light pulses that are highly tunable. By pumping the semiconductors silicon and gallium arsenide with visible light, the researchers were able to generate nanosecond pulsed thermal emission from their surfaces.

## Introduction

Short optical pulses have applications that range from telecommunication to ultrafast science to materials processing. Despite the relative maturity of pulsed sources in the visible and near-infrared spectral ranges, there is a deficiency of pulsed sources that operate in the mid infrared (wavelengths ~2–20 μm). Existing technologies have significant limitations; for example, mode locking of quantum cascade lasers is challenging and has resulted in sources with low power and limited tunability^1,2^, while down conversion of near-infrared pulses using nonlinear optics requires complex and expensive instrumentation^3,4^. Here, we explore an approach for generating short pulses in the mid infrared based on fast optically driven modulation of thermal emission.

According to Planck’s and Kirchhoff’s laws, the optical power that is thermally emitted by an object depends on both its temperature and emissivity^5^. The modulation of thermal emission can therefore be realized via dynamic changes in either of these two parameters. Fast modulation of temperature is in principle possible for emitters that have small volumes and, hence, small heat capacity. For example, electrical heating of carbon-nanotube films has been used to demonstrate thermal-emission modulation of up to 10 GHz^6^. Even faster temperature changes can be realized by decoupling the electronic and lattice temperatures: electrons can be driven far out of thermal equilibrium with phonons for a very short amount of time when pumped by a laser pulse^7^. Observations of hot-electron thermal emission have been reported in graphene^8^ and in metals^9^, although not in semiconductors.

While the temperature-modulation speed is inherently limited by the emitter’s heat capacity, the modulation of the emissivity has no such restrictions. Tunable emissivity can be achieved using materials whose optical properties change in response to external factors, such as the voltage^10–13^, optical field^14,15^, temperature^16–18^, and strain^19,20^. The fastest demonstration of emissivity modulation thus far used carrier-density tuning in a quantum-well-based gated thermal emitter, which resulted in modulation as fast as a few microseconds^13^. To the best of our knowledge, there has not been any experimental demonstration of the modulation of emissivity at or below the nanosecond time scale. In this work, we demonstrate pulse generation in the mid infrared based on fast modulation of the thermal emissivity of semiconductors at time scales of a few nanoseconds—three orders of magnitude faster than the previous record^13^. We also detected, for the first time, subnanosecond hot-carrier thermal emission from semiconductor emitters.

## Results

**Fig. 1:**
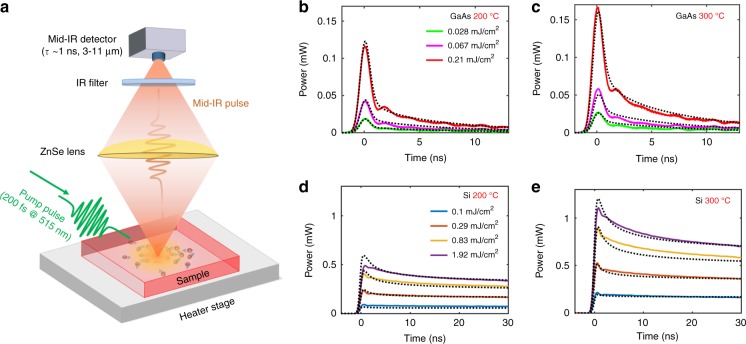
Experimental setup and the generation of thermally emitted pulses. **a** Schematic of the experiment. A flat and undoped semiconductor wafer was placed on a temperature-controlled stage. 200-fs laser pulses at 515 nm with a maximum energy of 500 µJ were incident onto the wafer at an angle of 45° with a repetition rate of 1 kHz. Absorption of the pump-laser pulses generates and heats free carriers in the undoped semiconductor, which results in thermal-emission pulses in the infrared. The thermal-emission pulses were collected with a zinc-selenide lens in the normal direction and then detected using a fast mid-infrared detector (response time ~1 ns, bandwidth of 3–11 µm). Optional infrared filters could be inserted before the detector to obtain spectral selectivity. The solid lines in **b**–**e** show calibrated thermal-emission pulses from GaAs and Si with different pump fluences from an emitting area of 14 mm^2^ toward a solid angle of 0.2 sr, at different stage temperatures. Note the difference in the *y* axis between **b**, **c** and **d**, **e**. The data in **b**–**e** was collected without any filters. The corresponding theoretical calculations are shown with dotted black lines

In this work, mid-infrared pulses are generated by rapidly modulating thermal emission from heated semiconductors using a visible pulsed laser (*λ* = 515 nm) (Fig. 1a). Undoped semiconductors are usually poor thermal emitters at photon energies below their band gap (e.g., silicon (Si) or gallium arsenide (GaAs) in the mid infrared^21^) but can become highly emissive via the presence of free carriers that can be generated via absorption of an above-gap optical pump pulse. As can be described by the Drude model^22^, an increase in free-carrier density increases the optical absorption, which results in an increased emissivity as expected from Kirchhoff’s law of thermal emission^23^. Note that we do not consider the case where the free-carrier density is so high that the material becomes metallic; in this extreme regime, the emissivity can be low even with high carrier density. After the pump is turned off, the free carriers recombine (this process takes a few nanoseconds for GaAs^21^ and a few microseconds for Si^21^), and the emissivity decreases. This mechanism of thermal-emission modulation via emissivity changes through free-carrier dynamics can thus be expected to generate thermal pulses with a duration that is mainly determined by the free-carrier lifetime. At the same time, for much shorter durations (typically a few picoseconds), the free carriers can be heated up to very high temperatures before they equilibrate with the lattice^8,^^9^. Thermal emission from these hot carriers (along with a change in the emissivity) is expected to have temporal widths of only a few picoseconds due to the rapid rise and fall in the free-carrier temperature.

Therefore, we expect ultrafast above-gap pumping of an undoped semiconductor to result in pulsed thermal emission with two different time scales (nanosecond and picosecond): the former from the change in free-carrier density, and the latter from hot-carrier dynamics. The spectrum and temporal width of the resulting emitted pulses should depend on both the pump characteristics and the semiconductor properties. To validate this idea, we conducted both experimental and theoretical investigations. In the following, we first present the experimental data and then build a model to explain the experimental results.

We tested our hypotheses using the experimental setup shown schematically in Fig. 1a. The emitter—a flat unpatterned wafer of intrinsic Si (thickness of 500 µm and background doping concentration of 2 × 10^11^ cm^−3^) or GaAs (thickness of 400 µm and background doping concentration of 1 × 10^12^ cm^−3^)—was placed on a temperature-controlled stage. The pump laser generated 200-fs pulses (Gaussian pulse duration and spatial profile) with a maximum energy of 500 µJ at a central wavelength of 515 nm, with a spot diameter of ~3.6 mm (Fig. S3, Supplementary Information). The repetition rate of the laser was 1 kHz, and thus the accumulation of heat across many pulses could be ignored (Section S5, Supplementary Information). The s-polarized pump laser was incident at an angle of 45°. The generated thermal pulses were collected by a zinc-selenide lens in the normal direction and were focused onto a fast and broadband AC-coupled mid-infrared detector (Boston Electronics PVI-4TE-10.6, response time of 1 ns with a bandwidth of 3–11 µm). For some experiments, infrared filters were inserted in front of the detector to obtain spectral selectivity. The output voltage from the detector was then recorded by an oscilloscope (Tektronix TDS7404B, 4 GHz, 20 GS/s).

The measurements without spectral filtering for GaAs and Si with different heater-stage temperatures and pump fluences are shown in Fig. 1b–e. Experimental data using higher pump fluence, achieved by focusing the pump laser, are shown in Fig. S1 of the Supplementary Information. The experiment using Si was performed with a higher pump fluence compared to the GaAs experiment because of the lower linear absorption coefficient of Si. Throughout this paper, we plot the estimated experimental power emitted toward the lens over the detector bandwidth of 3–11 μm; the conversions required to obtain these values are described in detail in Supplementary Information Section 3. We note that all emission data shown in this work are from AC-coupled measurements of the thermal emission. Any DC component of the measured thermal emission (e.g., from the nonzero emissivity of the sample stage underneath the sample and background emission from the optical setup) is suppressed at the high-pass filter in the preamplifier of the detector.

The thermal pulses detected from GaAs and Si show two features (Fig. 1b–e): a one-nanosecond peak that matches the temporal response of the detector is observed at the beginning of the pulse and is followed by a much slower decrease of the falling edge of the pulse. For GaAs, a relatively stronger one-nanosecond peak and a faster decay of the falling edge were observed. Comparing the experimental data at different temperatures, the magnitude of the falling edge of the thermal pulse for both GaAs and Si increases by roughly a factor of two when the sample-stage temperature is increased from 200 to 300 °C, which is consistent with the Stefan–Boltzmann law.

To better understand the experimental results and the process of thermal-pulse generation via free-carrier dynamics, we built a two-part model to calculate the time-dependent thermal emission from optically pumped semiconductors. The first part of the model characterizes the material response to an external optical pump pulse, and the second part calculates the corresponding thermal emission.

**Fig. 2:**
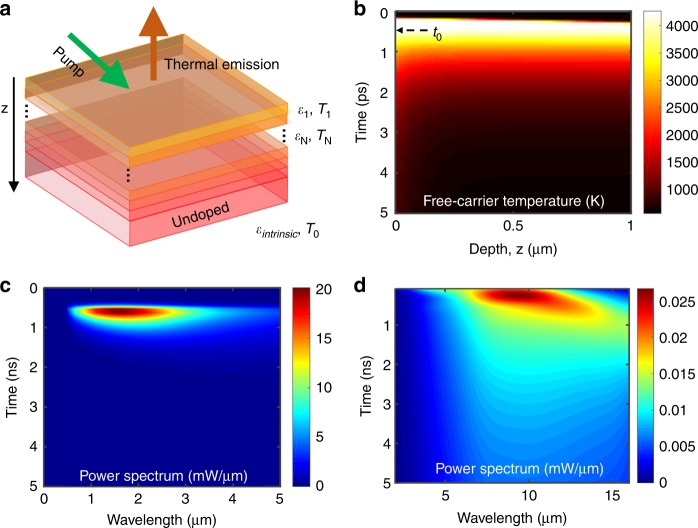
Theoretical model and simulations. **a** The optically pumped semiconductor wafer is modeled as a dynamic one-dimensional multilayer system, with each layer having different depth-dependent and time-dependent material properties *ε*(*z,t*) and temperature *T*(*z,t*) **b** Calculated free-carrier temperature for a 300 °C GaAs wafer with a pump fluence of 0.21 mJ/cm^2^. The corresponding power spectrum of the calculated emitted (**c**) picosecond thermal pulse from hot carriers and (**d**) nanosecond thermal pulse from the variation of free-carrier density. In (**d**), the time axis starts from 100 ps when the hot carriers are cooled down with the lattice, such that no hot carrier emission is included. The calculation assumes a 14-mm^2^ emitting area and a measurement solid angle of 0.2 sr, which matches the experiments

Following the absorption of an optical pump pulse, the free-carrier density and temperature inside of a bulk semiconductor are functions of both the depth and time. Therefore, we modeled the semiconductor wafer as a thin-film stack, with each layer having different time-dependent material properties and temperatures (Fig. 2a). To simulate the material response under the optical pump, we adapted the model from ref. ^24^ which is described in detail in Section 3 of the Supplementary Information. This model characterizes the interaction between a semiconductor and an external optical field. The semiconductor is assumed to have certain linear and nonlinear absorption. The free-carrier optical and thermal properties, as well as the lattice thermal properties, are also input parameters for this model. The interactions between light, carriers, and the lattice are simulated via coupled differential equations that we solve using the finite-difference time-domain method. The outputs of this portion of the model are the time-dependent and depth-dependent free-carrier density and temperature, as well as the lattice temperature.

Figure 2b shows the calculated free-carrier temperature as a function of depth for the top one micron of a GaAs wafer illuminated by a 200-fs pump pulse at 515 nm with a fluence of 0.21 mJ/cm^2^, which matches our experimental conditions. The center of the pump pulse reached the material surface at *t*_0_ = 0.5 ps. The corresponding carrier density and lattice temperature are shown in Fig. S6 of the Supplementary Information. The free carriers can be heated up to very high temperatures (~4000 K) for a very short time (<3 ps) due to the absorption of the visible-frequency pump. Heating of the free carriers occurs because the pump photon energy (2.41 eV) is much higher than the bandgap of GaAs (1.52 eV). This excess energy of the free carriers immediately after they are generated leads to a temperature that is much higher than that of the lattice. In the case of low pump power, the peak temperature *T* can be estimated using *T* ~ (*hf*−*E*_g_)/3*k*_B_ where *hf* is the pump photon energy, and *E*_g_ is the bandgap. In the case of high pump power, the peak temperature *T* will be higher than this simple estimate due to the effect of Auger heating (Section 4 of the Supplementary Information). After the pump, the free carriers quickly reach thermal equilibrium with the lattice through electron–phonon interactions.

Long after the pump pulse (hundreds of picoseconds; Fig. S7 of the Supplementary Information), the carriers are at the same temperature as the lattice. After this point, further changes of the free-carrier and lattice temperatures can be neglected because the lattice cooling time is on the order of microseconds (Section S5, Supplementary Information), which is much longer than our measurement time. Therefore, the only significant change to the material system is the free-carrier density, which is determined by diffusion and recombination:^24^

1 $$\frac{{\partial n}}{{\partial t}} = - \gamma n^3 - \frac{n}{{\tau _{\mathrm {c}}}} + D\Delta n$$

Here, *n*, *γ*, *τ*_c_, and *D* represent the carrier density, Auger recombination coefficient, free-carrier lifetime, and ambipolar diffusion coefficient, respectively.

The impact of the free carriers on the optical properties can be described using the Drude model:^22^

2 $$\varepsilon \left( {z,t} \right) = \varepsilon _{{\mathrm {intrinsic}}} - \omega _{\mathrm {p}}\left( {z,t} \right)^2/\left( {\omega ^2 + \frac{{i\omega }}{\tau }} \right)$$

where *ε* and *ε*_intrinsic_ are the permittivities of the photoexcited and intrinsic material, respectively, *τ* is the free-carrier relaxation time, and $\omega _{\mathrm {p}}(z,t) = \sqrt {n(z,t)e^2/(m\varepsilon _0)}$ is the plasma frequency. Additionally, *m* is the free-carrier effective mass, which is related to the electron and hole masses *m*_e_, *m*_h_ via $m^{ - 1} = m_{\mathrm {e}}^{ - 1} + m_{\mathrm {h}}^{ - 1\,}$^22^. Due to electron–electron and electron–lattice interactions, the free-carrier relaxation time depends on both the density^25,26^ and temperature^27,28^. To account for this effect, we used the Caughey–Thomas relation that was originally formulated for the density-dependent mobility in Si^29^ and has also been used for GaAs^25^ (Section S3, Supplementary Information).

The second part of the model calculates the thermal emission from the time-dependent system. Kirchhoff’s law cannot be used here directly because a single temperature cannot be defined: (1) the free-carrier and lattice temperatures can be different, and (2) both the free-carrier and lattice temperatures are depth-dependent (Figs. S5 and S6 of the Supplementary Information). Therefore, to calculate the thermal emission, we use a model that is based on the fluctuation–dissipation theorem (FDT) and dyadic Green’s functions from ref. ^30^ (Section 3, Supplementary Information). Thermal emission originates from the thermally induced random currents inside an object. The power spectrum of the random current sources is given by the FDT, and Green’s functions can used to calculate the corresponding electromagnetic fields from these sources. In our one-dimensional thin-film stack, Green’s function can be obtained via the scattering-matrix approach^30^. We note that the recently developed local Kirchhoff law^31^ could have been used instead.

Figure 2c, d shows the calculated time-dependent power spectrum of the picosecond (due to the rapid temperature change of the hot carriers as well as the carrier-density dynamics) and nanosecond (due to carrier-density dynamics only, no hot-carrier contribution) features of the thermal emission from a GaAs wafer in the normal direction with a solid angle of 0.2 sr and an emitting area of 14 mm^2^. The hot-carrier thermal pulse plotted in Fig. 2c is only a few picoseconds long due to the small hot-carrier lifetime. The spectral peak of this pulse is in the near-infrared region (~1.5 µm) due to the high temperature of the carriers (Fig. 2b). The nanosecond thermal pulse plotted in Fig. 2d is much longer because the free-carrier lifetime is three orders of magnitude larger than the hot-carrier lifetime (nanoseconds vs. picoseconds). The spectral peak of the nanosecond thermal pulse is in the mid infrared (shifting from 8 µm to longer than 10 µm with increasing time), a result of the much-lower temperature of the free carriers when they are thermally equilibrated with the lattice (~600 K), compared to immediately after the pump (~4000 K).

We performed calculations using experimental parameters (Section 4, Supplementary Information), and the results are plotted using dotted black lines in Fig. 1b–e. The material parameters for Si and GaAs were chosen from reasonable values in the literature, with several of the parameters selected to achieve good agreement with the experiments (Section 3, Supplementary Information). The calculations reproduce our experimental features quite well. With the help of the model, we can better understand our experimental results. The hot-carrier thermal emission results in an ultrafast pulse of only a few picoseconds wide (Figs. 2c and S9a), which leads to the one-nanosecond-duration peak experimentally observed in Fig. 1b–e. The broader pulse in the experiment is due to the 1-ns response time of our detector. The much-slower decrease of the emission signal after the short hot-carrier pulse is due to the reduction of the photogenerated free-carrier density and thus the emissivity. GaAs has a free-carrier lifetime of a few nanoseconds, and thus the detected thermal-emission signal decreases to a very low value within ~10 ns (Figs. 1b, c and S1a, b). The decay rate of the emission from Si is much lower than from GaAs (Figs. 1d, e and S1c, d); Si has a much longer free-carrier lifetime (a few microseconds^21^). Within a temporal window of 30 ns, diffusion and Auger recombination are the primary mechanisms for changes to the carrier density for Si. Indeed, an increasing decay rate of the measured thermal-emission signal with increasing pump fluence is observed here, which demonstrates the impact of Auger recombination. Note that the magnitudes of the initial hot-carrier emission pulses are very similar for Si and GaAs but, at longer times, the emissivity of the GaAs wafer decreases faster than the emissivity of Si, which results in more thermal-emission power from the carriers that are now in thermal equilibrium with the lattice. The difference in emissivity is due to differences in the diffusion coefficient, the Auger recombination rate, and the free-carrier scattering time between the two materials (Sections S3 and S4 of the Supplementary Information).

Because thermal emission depends on the temperature, the temperature of the stage-heated wafer should have a significant impact on the emitted pulses. In Figures 1b, c and S1a, b, the amplitude of the pulse that results from the change in emissivity (the slower decay) increases by approximately a factor of 2, which is in agreement with the Stefan–Boltzmann law. The one-nanosecond peak amplitude, however, does not change significantly with the stage temperature, because this peak comes from hot-carrier thermal emission. As shown in our calculation (Fig. 2b), the free-carrier temperature is mainly determined by the pump photon energy and the bandgap rather than the wafer temperature.

**Fig. 3:**
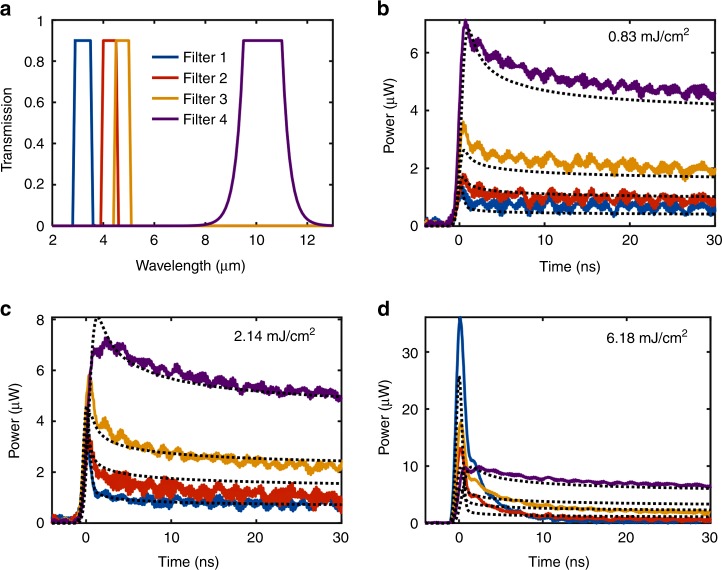
Resolving spectral features using infrared filters. **a** Transmission spectra of the infrared filters used in our experiment. **b**–**d** Solid lines: experimentally calibrated thermally emitted power from a 1.4 mm^2^ emitting area into a solid angle of 0.2 sr, using the filters, from a Si wafer at 300 °C with a pump fluence of (**b**) 0.83, (**c**) 2.14, and (**d**) 6.18 mJ/cm^2^. The corresponding theoretical calculations are shown using dotted black lines

As seen from Fig. 2d, the spectrum of the thermal pulse changes with time due to the time-dependent emissivity and free-carrier temperature. To determine the spectral composition of the thermal pulses, we performed measurements with four infrared transmission filters with different passbands placed between the sample and the detector (Fig. 1a). The transmission windows for the filters are centered at approximately 3, 4, 5, and 10 µm (Fig. 3a). The thermal pulses from a 300 °C Si wafer (from an emitting area of 1.4 mm^2^ into a solid angle of 0.2 sr) were measured through each filter with three different pump fluences (0.83, 2.14, and 6.18 mJ/cm^2^) and are plotted using solid lines in Fig. 3b–d. The corresponding theoretical calculations are plotted using dotted black lines. Similar experimental data but using GaAs are shown in Fig. S2 in the Supplementary Information.

When the pump fluence is low (Fig. 3b, 0.83 mJ/cm^2^), the measurements with filters indicate that the emitted pulse has much more power at longer wavelengths. This finding is expected, since the hot-carrier thermal emission is very weak at such low pump power, and most of the thermal emission comes from carriers at the temperature of the wafer (300 °C), with the corresponding peak thermal-emission wavelength in the mid-infrared region. When the pump fluence is increased (Fig. 3c, 2.14 mJ/cm^2^), a relative increase in the signal through the shorter-wavelength filters (3, 4, and 5-µm filters) is seen at the beginning of the pulse near *t* = 0 ns, which indicates an increased contribution from emitting components at a higher temperature than the wafer and sample stage. This increase becomes obvious for the highest pump fluence (Fig. 3d, 6.18 mJ/cm^2^): the emission measured through the 3-µm filter is the highest and is much higher than that through the 10-μm filter. This observation is evidence of thermal emission from hot carriers. The relatively larger increase in the hot-carrier emission vs. the pump power is due to Auger heating (Section S4 of the Supplementary Information). Note that due to the relatively slow response of the detector (~1 ns), the measured thermal emission near *t* = 0 ns in Fig. 3c, d has contributions from both hot carriers and carriers that are in thermal equilibrium with the lattice. After the pump pulse, the hot carriers quickly reach thermal equilibrium with the lattice (Fig. S7, Supplementary Information); thus, the emission measured through shorter-wavelength filters decays very quickly (Fig. 3d). Long after the pump, the strongest emission is through the 10-µm filter.

## Discussion

One can view this process of generating mid-infrared pulses from pulses in the visible range as an unconventional type of frequency conversion, which differs substantially from traditional nonlinear processes. In our approach, the conversion efficiency from the visible to the mid infrared depends on many factors, such as the pump power and wavelength, the sample temperature, and the material system used. Defining the conversion efficiency as the total energy in the infrared pulse collected at our detector (i.e., over a solid angle of 0.2 sr) divided by the total energy in the visible pump pulse, we find that the conversion efficiency for our Si emitter is much higher than that for our GaAs emitter due to the much-longer pulse tail, which results from the longer free-carrier lifetime. For example, we obtain a conversion efficiency of ~2 × 10^−8^ from our GaAs emitter at 300 °C with a pump fluence of 0.21 mJ/cm^2^, and 6 × 10^−6^ from our Si emitter at 200 °C with a pump fluence of 2.14 mJ/cm^2^. The actual conversion efficiencies (i.e., over a solid angle of 2π sr) are ~6 × 10^−7^ and 2 × 10^−5^, respectively. These values of conversion efficiency are not very high, but many approaches can, in principle, be used to increase them considerably; see Supplemental Section 7 for more discussion.

The bandwidth of the infrared pulses is quite large and is mostly independent of the bandwidth of the input pulse. The resulting broadband pulses can be sent through bandpass filters to yield more-narrowband pulses at any wavelength in the mid infrared, albeit with a reduction in the power. Such tunability is difficult to attain using existing pulse-generation techniques, such as mode-locking of quantum cascade lasers or downconversion. Note that because the thermally emitted pulse is strongly chirped, a narrowband filter will reduce the pulse duration rather than increase it, which is the case for Fourier-limited pulses.

To conclude, we experimentally demonstrated the generation of few-nanosecond thermal pulses via emissivity modulation of semiconductor surfaces using an ultrafast visible pump. We also observed subnanosecond thermal emission from hot carriers in semiconductors. Although the resulting pulse energies are limited by Planck’s law for a given temperature, this method of generating mid-infrared pulses is quite simple and has substantial tunability compared to existing approaches. In addition to the use of in-line optical filters to achieve wavelength tunability, the spectral and temporal features of this thermal pulse can also be tuned by choosing different combinations of the pump laser and semiconductor material, and the bandwidth and directionality can be engineered using various nanophotonic design structures^5^, for example, using gratings^32^ or by patterning the pump-laser beam profile^15^. We anticipate that the generation of infrared pulses over a wide range of wavelengths using modulated thermal emission will lead to applications in optical communications and various sensing and spectroscopy techniques.

## Materials and methods

The samples are flat unpatterned intrinsic Si (thickness of 500 µm and background doping concentration of 2 × 10^11^ cm^−3^) and GaAs (thickness of 400 µm and background doping concentration of 1 × 10^12^ cm^−3^) wafers. The pump lasers at 515 nm are frequency-doubled from a 1030-nm femtosecond laser system, which has a repetition rate of 1 kHz. The pulse at 515 nm has a Gaussian spatial profile with a spot diameter of ~3.6 mm, a Gaussian pulse shape with a duration of 200 fs, and a maximum energy of 500 µJ. The detector is from Boston Electronics (PVI-4TE-10.6), and has a response time of 1 ns with a bandwidth of 3–11 µm. The detection efficiency was simulated with Zemax raytracing software. More details of the experiments and modeling can be found in the Supplementary Information.

## Supplementary information

Supplementary Information
