# Supplementary material for: Nanosecond mid-infrared pulse generation via modulated thermal emissivity
Source: Light Sci Appl. 2019 Jun 5;8:51. doi: 10.1038/s41377-019-0158-6 (PMC6548774; doi:10.1038/s41377-019-0158-6)
Supplement: Supplementary file 1 — Supplementary Information [file 41377_2019_158_MOESM1_ESM.docx]

Supplementary Information:

Nanosecond mid-infrared pulse generation via modulated thermal emissivity

Yuzhe Xiao^1^, Nicholas A. Charipar^2^, Jad Salman^1^, Alberto Piqué^2^ and Mikhail A. Kats^1,3,4,*^

*^1^Department of Electrical and Computer Engineering, University of Wisconsin-Madison, Madison, Wisconsin, USA 53706*

*^2^ Naval Research Laboratory, 4555 Overlook Ave. SW, Washington, DC 20375, USA*

*^3^Materials Science and Engineering, University of Wisconsin-Madison, Madison, Wisconsin, USA 5370*

*^4^Department of Physics, University of Wisconsin - Madison, Madison, Wisconsin 53706, USA*

**Email address:* [*mkats@wisc.edu*](mailto:xiao5@wisc.edu)

**S1: Additional experimental data**

We measured pulsed thermal emission from silicon (Si) and gallium arsenide (GaAs) wafers with a higher pump fluence than that shown in the manuscript by focusing the pump laser spot size down to 1.4 mm^2^, which is 10% of the original spot area. This increases the fluence by a factor of 10 for the GaAs case. For Si, we found that 10$\times$ the fluence from Fig. S1 resulted in damage, so we reduced the laser power somewhat; the resulting fluences are in the legend of Fig. S1. In Fig. S1, we plot the experimentally calibrated emitted power, radiated into a solid angle of 0.2 sr within the detector bandwidth of 3-11 μm, for stage temperatures of 200 and 300 °C. The corresponding calculations from our theoretical model are plotted using dotted black lines.

**
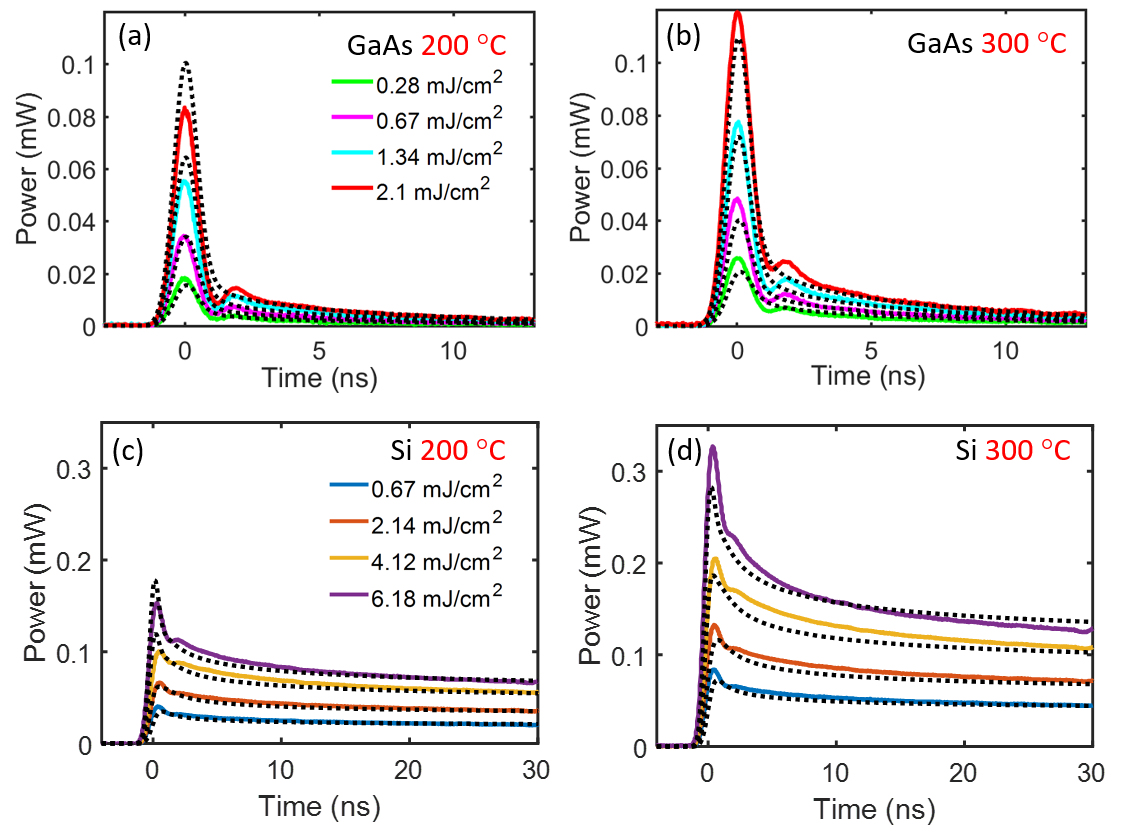
**

Figure S1. Solid lines: experimentally calibrated thermal-emission power from polished (a, b) GaAs and (c, d) Si from an emitting area of 1.4 mm^2^ into a solid angle of 0.2 sr, at stage temperatures of 200 and 300 °C, for higher pump fluences than those shown in the main text. The corresponding theoretical calculations are shown with black dotted lines.

We also used filters to obtain spectral information about the thermal pulses from the GaAs wafer, just like for Si in the main text (Fig. 3). Figure S2 shows the calibrated experimental emitted power passing through each filter (Fig. 3(a) in the main text) from a 300 °C GaAs wafer with an emitting area of 1.4 mm^2^ into a solid angle of 0.2 sr, for pump fluence of 0.67, 1.34, and 2.1 mJ/cm^2^. The corresponding calculations from theoretical model are also shown.

**
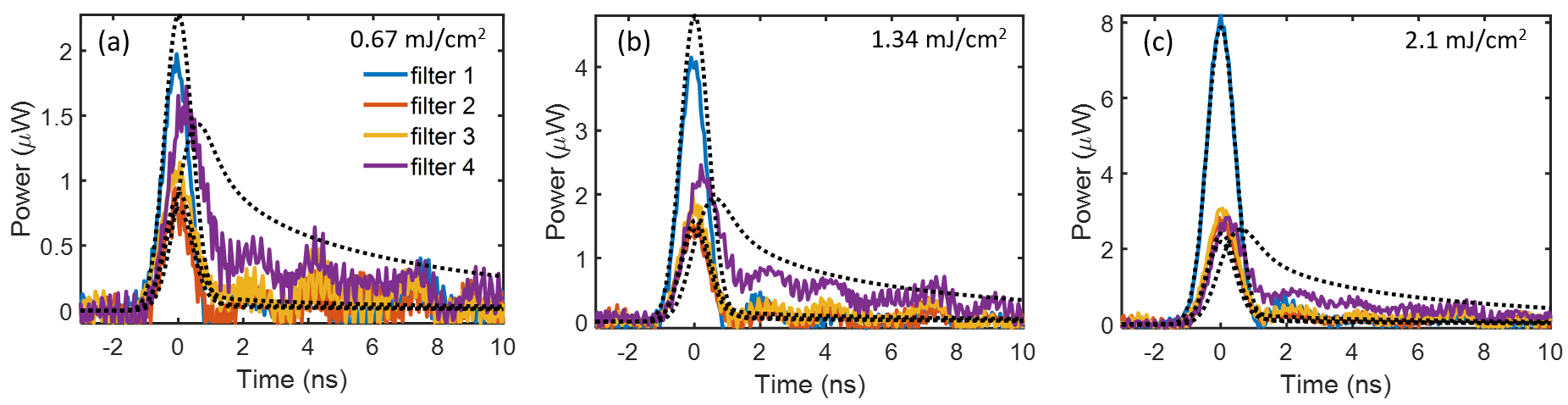
**

Figure S2. Solid lines: Measured emitted power (from a 1.4 mm^2^ emitting area into a solid angle of 0.2 sr) through different filters (shown in Fig. 3(a), with passbands near 3, 4, 5 and 10 μm for filter 1, 2, 3 and 4, respectively) from a 300 °C GaAs wafer with pump fluence of (a) 0.67, (b) 1.34, and (c) 2.1 mJ/cm^2^. The corresponding theoretical calculations are shown using dotted black lines.

**S2: Additional details of the experimental setup**

Here, we present additional details of the experiment setup, including the pump laser profile and an estimation of the detection efficiency (the fraction of power that is detected divided by the total power passing through our collection lens).

The intensity profile of the 515-nm pump laser pulse is shown in Fig. S3, which can be approximated by a Gaussian profile with an effective diameter (width of a beam that is 4 times σ, where σ is the standard deviation of the intensity distribution) of about 3.6 mm. When the beam is incident at an angle of 45°, the corresponding illuminated (and hence emitting) area on the sample is about 14 mm^2^.

**
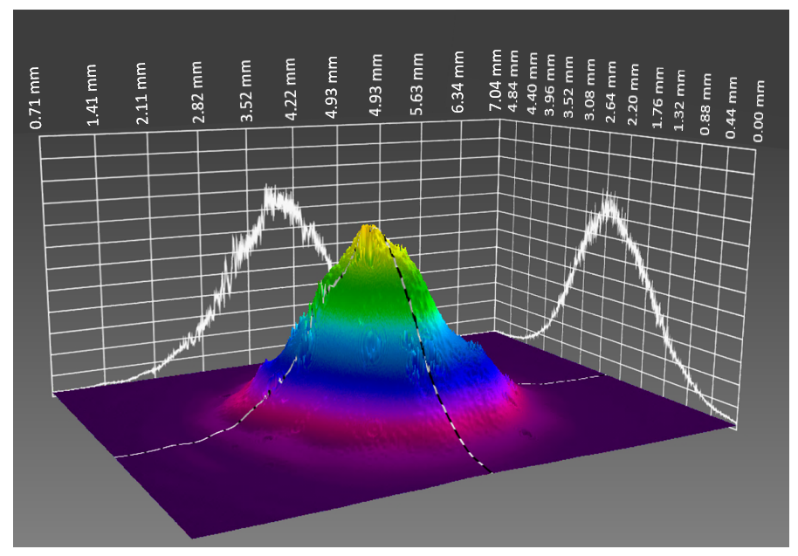
**

Figure S3. Measured intensity profile of the 515-nm pump pulse laser at normal incidence (Spiricon SP620U). The laser spot can be approximated by a Gaussian with an effective diameter of about 3.6 mm.

We used a single zinc selenide (ZnSe) lens (diameter of 25.4 mm, focal length of 25 mm) to collect and focus the thermal emission onto the detector. The lens was placed about 60 mm away from the sample in the normal direction, corresponding to a collection solid angle of about 0.2 sr. The two sample emitting areas were 14 and 1.4 mm^2^ for the unfocused and focused case, respectively. The active area of the detector is 0.25 mm^2^ (Boston Electronics PVI-4TE-10.6, 0.5 mm $\times$ 0.5 mm).

Since only a fraction of the emitted power that passes through the lens is incident on the active area of the detector, the precise emitted power was not directly measurable. To estimate the actual power emitted by the sample into a certain solid angle, we performed ray-tracing simulations using Zemax OpticsStudio to approximate the total power passing through the lens (Fig. S4). Figure S4(a) shows the layout of the case with the unfocused pump. For the unfocused case, the emitting source area is 14 mm^2^. In this case, based on simulations, only about 3.5% of emitted power was incident on the detector element [Fig. S4 (b)]. In the focused case, the emitting area is smaller, and more-easily images onto the active area of the detector, resulting in 15% of the emitted light being focused onto the detector element [Fig. S4 (c)]. This increase in collection efficiency matches our experimental observations.

**
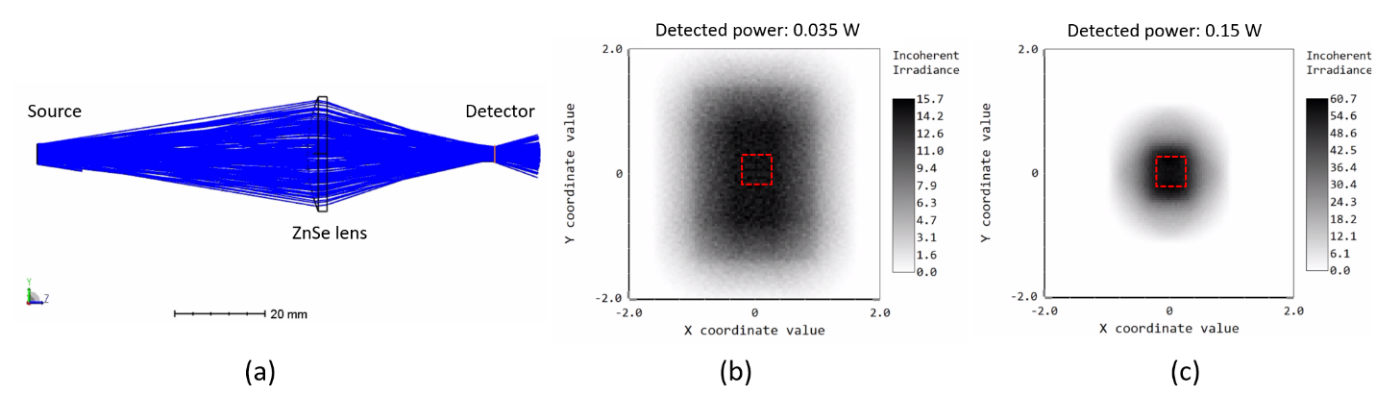
**

Figure S4. (a) Layout of the ray-tracing simulation using Zemax OpticsStudio for the unfocused case: the source has an emitting area of 14 mm^2^ and was placed 60 mm away from the lens. The source is about 1.4 times larger in in the y-direction than x-direction, to mimic the oval shape of the pump laser spot on the sample. The detector has an active area of 0.25 mm^2^ and was placed about 40 mm after the lens. (b) Detector analysis for the unfocused case: about 0.035 W of power was incident on the detector element (rectangular area) assuming 1 W emitting from the source towards the lens (corresponding to a solid angle of about 0.2 sr). (c) Detector analysis for the focused case: the source area is reduced from 14 to 1.4 mm^2^. About 0.15 W of power was incident on the detector element (rectangular area), assuming 1 W emitting from the source towards the lens.

Because the actual power emitted in a given solid angle is more fundamental than the power detected by our finite-size detector element, the pulse power we report in all experimental figures (Figs. 1, 3, S1 and S2) is the estimated power emitted toward the lens in the detector bandwidth of 3 to 11 μm, which is the actual power detected divided by the corresponding fraction ratio estimated from ray tracing simulations. The detected power is obtained from the detected voltage through the detector voltage responsivity (1.4$\times{10}^{4}$ V/W).

**S3: Theoretical modeling**

This section shows more details of the model we used to calculate the temporal and spectral features of pulsed thermal emission, including the details of light-matter interaction inside our two semiconductors, the scattering rates of the free carriers used in the Drude model, and the method of calculating thermal emission from an object with spatially and temporally varying temperature and optical properties.

We adopted the model from Ref. [S1] to calculate the electron-hole pair (free-carrier) density and temperature, as well as the lattice temperature when a semiconductor is excited by an optical pulse. More specifically, the following equations are solved:

$\frac{\partial n}{\partial t}=\frac{\alpha I(z,t)}{hv}+\frac{\beta I^{2}(z,t)}{2hv}-\gamma n^{3}-\frac{n}{\tau_{c}}+\theta n-\nabla\cdot\boldsymbol{J}$ (S1)

$\boldsymbol{J}=-D\left( \nabla n+\frac{n}{2k_{B}T_{e}}\nabla E_{g}+\frac{n}{2T_{e}}\nabla T_{e} \right)$ (S2)

$\boldsymbol{W}=\left( E_{g}+4k_{B}T_{e} \right)\boldsymbol{J-(}k_{e}\boldsymbol{+}k_{h}\boldsymbol{)}\nabla T_{e}$ (S3)

$$C_{e-h}\frac{\partial T_{e}}{\partial t}=\left( \alpha+\Theta n \right)I\left( z,t \right)+\beta I^{2}\left( z,t \right)-\frac{C_{e-h}}{\tau_{hot}}\left( T_{e}-T_{l} \right)$$

$-\nabla\cdot\boldsymbol{W}-\frac{\partial n}{\partial t}\left( E_{g}+3k_{B}T_{e} \right)-n(\frac{\partial E_{g}}{\partial T_{l}}\frac{\partial T_{l}}{\partial t}+\frac{\partial E_{g}}{\partial n}\frac{\partial n}{\partial t})$ (S4)

$C_{l}\frac{\partial T_{l}}{\partial t}=\nabla\cdot\left( k_{l}\nabla T_{l} \right)+\frac{C_{e-h}}{\tau_{hot}}\left( T_{e}-T_{l} \right)$ (S5)

$\frac{\partial I}{\partial z}=-\left( \alpha+\Theta n \right)I\left( z,t \right)-\beta I^{2}\left( z,t \right)$ (S6)

The free-carrier density $n$ is solved using Eq. S1, where $I$ is the light intensity, $\alpha,\beta$ are linear and two-photon absorption coefficients, $h$ is Planck’s constant, $v$ is pump light frequency, $\gamma$ is the Auger recombination coefficient, $\tau_{c}$ is the free-carrier lifetime, and $\theta$ is the impact ionization coefficient. The free-carrier current $\boldsymbol{J}$ and ambipolar energy $\boldsymbol{W}$ are defined using Eqs. S2-3, where $T_{e},T_{l}$ are the free-carrier and lattice temperatures, $E_{g}$ is the bandgap, $k_{e},k_{h}$ are the thermal conductivity of electrons and holes. The free-carrier and lattice temperature are solved using Eqs. S4-5, where $C_{e-h}, C_{l}$ are the heat capacitances for electron-hole pairs and the lattice, and $\tau_{hot}$ is the lifetime of the hot carriers. Changes to light intensity $I$ is described by Eq. S6, where $\Theta$ is the free-carrier absorption cross-section. Note that $n, I,\boldsymbol{J,W}, T_{e}, \mathrm{and} T_{l}$ are all depth- ($z$) and time-dependent, where $z$ is the distance into the material from the surface. Solving these equations using a one-dimensional finite-difference time-domain (FDTD) algorithm can yield the depth- and time-dependent free-carrier density, free-carrier temperature, and the lattice temperature.

Table S1 summarizes all the parameters that we used when solving Eqs. S1-6 for Si and GaAs. Most of the model parameters were chosen according to the reported literature values. Some of the parameters ($\tau_{hot}$,$\tau_{c}$,$\gamma$) are chosen to have the best agreement with experimental results, and are close to the reported literature values. Since the model was built for Si [S1], not all of the parameters are readily available for GaAs, which is why we had to make some assumptions below. This also might make our model less reliable for GaAs.

Table S1: Model parameters

| Properties | Silicon | Gallium Arsenide |
| --- | --- | --- |
| $k_{l}$[W/(cm$\cdot$K)] | $1585T_{l}^{-1.23}$ [S1] | $686T_{l}^{-1.25}$ [S2] |
| $C_{l}$(J/cm^3^) | 1.978 $+$ 3.54 $\times{10}^{-4}T_{l}-T_{l}^{-2}$ [S1] | 1.72 $+$ 2.8 $\times{10}^{-4}T_{l}-1.87 \times{10}^{3}T_{l}^{-2}$ [S3] |
| $k_{e}$ [eV/(s$\cdot$A$\cdot$K)] | $-3.47 \times{10}^{8} +4.45 \times{10}^{6}T_{e}$ [S1] | $-2.1 \times{10}^{9} +2.7 \times{10}^{7}T_{e}$ [S1]^*^ |
| $\tau_{hot}$ (fs) | $500 \cdot(1+n/6 \times{10}^{20} \mathrm{cm}^{-3})$ [S4]–[S6] | $750 \cdot(1+n/6 \times{10}^{20} \mathrm{cm}^{-3})$ [S7][S8] |
| $\gamma$ (cm^6^/s) | $10 \times{10}^{-31}$ [S1][S9] | $11 \times{10}^{-30}$ [S10] |
| $\tau_{c}$ (ns) | 1000, Appendix G of Ref. [S11] | 10, Appendix G of Ref. [S11] |
| $\theta$ (s^-1^) | $3.6 \times{10}^{10} exp(-1.5E_{g}/k_{B}T_{e})$ [S1] | (assumed to be negligible) [S12] |
| $D_{0}$ (cm^2^/s) | $18 \cdot(T_{rm}/T_{l})$ [S1] | $20 \cdot(T_{rm}/T_{l})$ [S13] |
| $E_{g}$ (eV) | $1.16-7.02 \times\frac{{10}^{-4}T_{l}^{2}}{T_{l}+1108}-1.5 \times{10}^{-8}n^{1/3}$ [S1] | $1.52-8.87 \times\frac{{10}^{-4}T_{l}^{2}}{T_{l}+572}-2 \times{10}^{-11}n^{-1/2}$ [S14], [S15] |
| $\alpha$ (cm^-1^) | $3.42 \times{10}^{4}$ [S16] | $7.81 \times{10}^{4}$ [S17] |
| $\beta$ (cm/GW) | (assumed to be negligible) [S1] | 3.6 [S18][S19]^+^ |
| $\Theta$ (cm^2^) | $5.1 \times{10}^{-18} \cdot(T_{l}/T_{rm})$ [S1] | $5.5 \times{10}^{-18} \cdot(T_{l}/T_{rm})$ [S20]^o^ |
| $m$ (free electron mass) | 0.15 [S11] | 0.058 [S11] |

^*^ Value derived from Si, assuming the thermal conductivity of electrons is proportional to its mobility.

^+^ Using the experimental value near 1 $\mu$m from ref. [S19] , and the scaling rule from ref. [S18] to extrapolate the value for 0.515 $\mu$m.

^o^ Using the experimental value near 1 $\mu$m.

The following expression was used for the scattering time of free carriers in the Drude model [S21]:

$\tau=\frac{\tau_{max}-\tau_{min}}{1+{(n/N_{0})}^{\alpha}}+\tau_{min}$. (S7)

Note that immediately after the pump, the carriers have energy much higher than the bandgap (hot carriers), which increases their interaction with the lattice, thereby increasing their scattering rate [S22], [S23]. Due to the additional scattering from the lattice, values for the time constants for the hot carriers are an order of magnitude smaller than those for carriers that have cooled down by reaching thermal equilibrium with the lattice [S22], [S23]. The parameters used in Eq. S7 in our model are chosen according to literature values [S24], [S25] and are shown in Table S2.

Table S2: Model parameters for the free-carrier scattering time

| Properties | Silicon | | Gallium Arsenide | |
| --- | --- | --- | --- | --- |
|  | Hot carriers | Cold carriers | Hot carriers | Cold carriers |
| $\tau_{max}$(fs) | 4 | 200 | 7.5 | 375 |
| $\tau_{min}$(fs) | 0.4 | 20 | 0.1 | 5 |
| $N_{0}$ (1/cm^3^) | 0.5$\times{10}^{17}$ | 0.5$\times{10}^{17}$ | $1 \times{10}^{17}$ | $1 \times{10}^{17}$ |
| $\alpha$ | 0.7 | 0.7 | 0.2 | 0.2 |

To calculate the thermal emission from a system with depth- and time-dependent temperature and permittivity distributions, we used a model from Ref. [S26]. Consider the thermal radiation emitted by an arbitrary object with position-dependent temperature $T(\boldsymbol{r'})$ and permittivity $\epsilon(\boldsymbol{r'})$ evaluated at an observation point $\boldsymbol{r}$**.** The position-dependent temperature results in a position-dependent distribution of random currents $\boldsymbol{j}(\boldsymbol{r'}, t)$ and their Fourier transform, $\boldsymbol{j}(\boldsymbol{r'}, \omega)$ throughout the object. The power spectrum of these random currents is given by the fluctuation-dissipation theorem (FDT). The simplified FDT, valid for isotropic linear materials described by dielectric constant $\epsilon(\omega)$, is (Chapter 14 of Ref. [S27]):

$\frac{1}{\omega Im[ \epsilon_{0}\epsilon(\omega)]}\pi<j_{\alpha}(\boldsymbol{r}_{\boldsymbol{1}},\omega)j_{\beta}^{*}(\boldsymbol{r}_{\boldsymbol{2}},\omega')>=$ $\Theta\left( \omega,T \right)\delta(\boldsymbol{r}_{\boldsymbol{1}}-\boldsymbol{r}_{\boldsymbol{2}})\delta(\omega-\omega')\delta_{\alpha\beta}$, (S8)

where $\alpha$ and $\beta$ refer to different Cartesian components of the current, $\Theta\left( \omega,T \right)=h\omega/(e^{\frac{\hbar\omega}{k_{B}T}}-1)$ is the mean (expected) energy in a particular state with frequency $\omega$ given temperature $T$, $\epsilon_{0}$ is the vacuum permittivity, $\delta$ is the Dirac delta function, and $\delta_{\alpha\beta}$ is the Kronecker delta. The deltas express that the fluctuating currents at different positions, frequencies, and orthogonal directions are uncorrelated. The ensemble average (<…>) is the average over all combinations of possible random currents.

The averaged radiative intensity at $\boldsymbol{r}$, $<\boldsymbol{S}\left( \boldsymbol{r},\omega\right)>,$ where $\boldsymbol{S}$ is the Poynting vector, is related to the averaged spectral power of the random current source $<j_{\alpha}\left( \boldsymbol{r},\omega\right)j_{\beta}^{*}\left( \boldsymbol{r},\omega\right)>$, which is given by FDT. Therefore, in order to determine the radiated intensity, one just needs to find the relation between the source current and the corresponding fields. For a current density at some point in space, $\boldsymbol{j}(\boldsymbol{r}^{\boldsymbol{'}}, t)$, the resulting fields at $\boldsymbol{r}$ is given by the dyadic Green’s function of the system $\bar{G}\left( \boldsymbol{r,}\boldsymbol{r}^{'} \right)$ (Chapter 14.7 of Ref. [S28]):

$\boldsymbol{h}(\boldsymbol{r,\omega})=\iiint\boldsymbol{j}\left( \boldsymbol{r}^{'},\omega\right)\cdot\bar{G}\left( \boldsymbol{r,}\boldsymbol{r}^{'},\omega\right)dv'$, (S9)

where $\boldsymbol{h}$ is the electric or magnetic field for the corresponding electric and magnetic Green’s function. The averaged radiative intensity $<\boldsymbol{S}\left( \boldsymbol{r},\omega\right)>$ is determined both by the Green’s function $\bar{G}\left( \boldsymbol{r,}\boldsymbol{r}^{'} \right)$and spectral power of the random currents $<j_{\alpha}\left( \boldsymbol{r},\omega\right)j_{\beta}^{*}\left( \boldsymbol{r},\omega\right)>$. In our one-dimensional thin film stack, the Green’s function can be obtained via the scattering matrix [S26]. The calculation based on FDT and Green’s function is performed for different times so that we finally have time-dependent thermal emission.

**S4: Results from the model**

Figure S5 shows the calculated free-carrier density ($n$), carrier temperature ($T_{e}$) and lattice temperature ($T_{l}$) for a Si wafer on a temperature stage at 300 °C, illuminated by a 200-fs Gaussian pump pulse with two different fluences: 0.1 and 1.92 mJ/cm^2^. The pump pulse center arrives at the material surface at $t_{0}=$0.5 ps. As shown in Fig. S5 (a), a peak carrier density about 2$\times{10}^{19}$ cm^-3^ is generated near the surface with a low pump fluence of 0.1 mJ/cm^2^. The carrier density shows an exponential decay into the material, with most of the carriers well confined to an approximately 1-$\mu$m-thick layer. After the pump (t > 1 ps), the carrier density remains almost unchanged because of the long free-carrier lifetime. When the pump fluence is increased to 1.92 mJ/cm^2^ [S5 (d)], the peak carrier density increases to 4$\times{10}^{20}$ cm^-3^. However, unlike in the lower-intensity pump scenario, the carrier density, particularly near the surface ($z$ $=0$ $\mu$m), decreases quickly after the pump, due to Auger recombination.


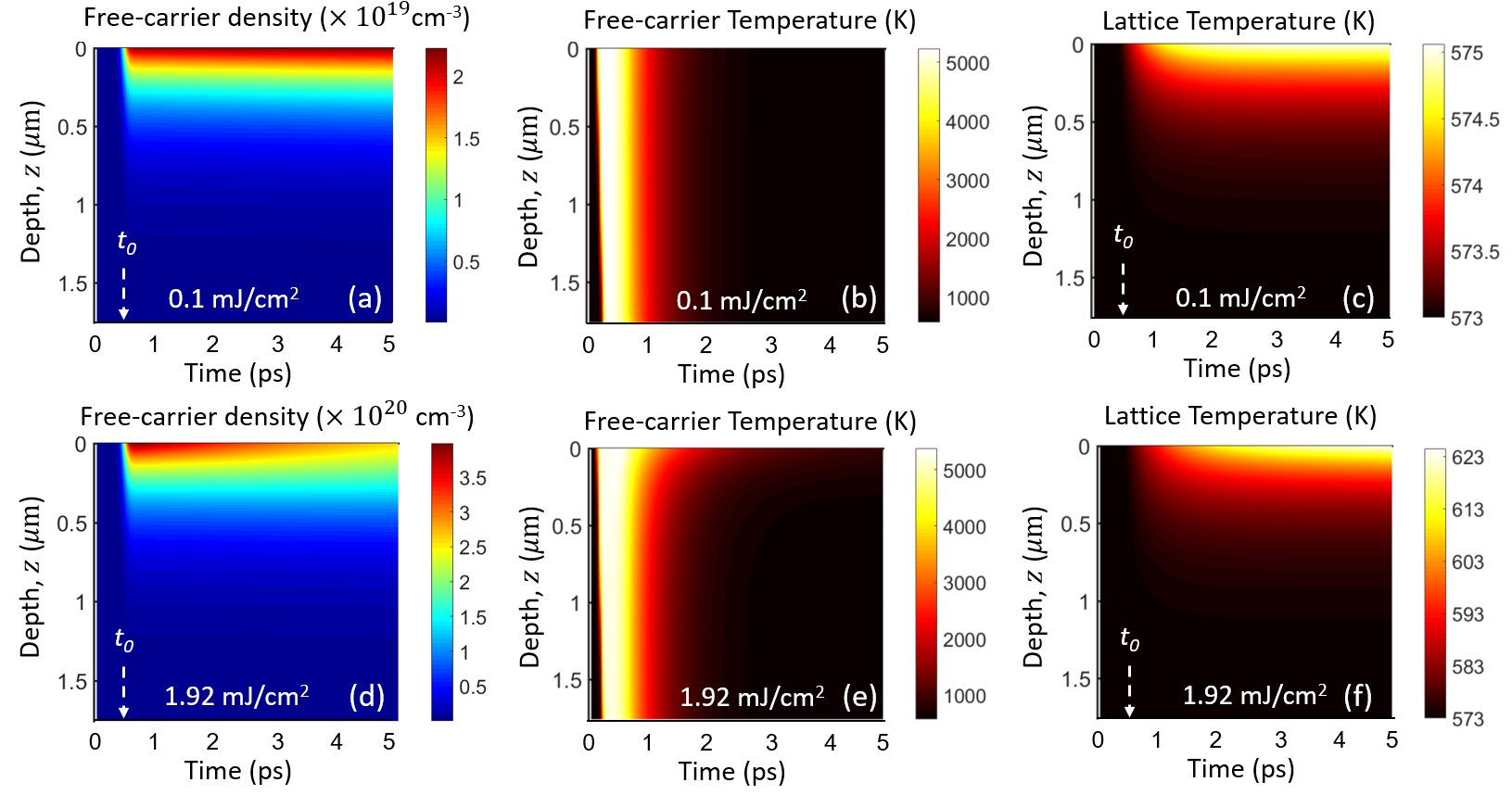


Figure S5. Calculated time- and depth-dependent free-carrier density (a and d), free-carrier temperature (b and e), and lattice temperature (c and f) for a 300 °C (537 K) Si wafer excited by a 200-fs Gaussian pump at 515 nm with fluence of 0.1 (top) and 1.92 mJ/cm^2^ (bottom).

As shown in Fig. S5(b, e), the free carriers are heated to a very high temperature, far out of equilibrium with the lattice immediately after the pump. The peak free-carrier temperatures are at about 5000 K for both pump fluences [Fig. S5 (b, e)], which agrees with the estimated peak temperature of 4830 K using $T \sim(hf-E_{g})/3k_{B}$, where $hf-E_{g}$ is the difference between the pump photon energy and the band gap, and 3$k_{B}T$ is the corresponding thermal energy of the carrier. Due to the effect of Auger heating [S29], the peak free-carrier temperature is higher and the free-carrier cooling time is longer for the higher pump case (e), especially near the material surface ($z$ ~ 0 $\mu$m). For both (c) low and (f) high pump fluences, the lattice temperature increases due to the interaction with the hot carriers. Heating of the lattice is negligible for a pump fluence of 0.1 mJ/cm^2^, while the lattice temperature can be increased by more than 50 K when the pump fluence is raised to 1.92 mJ/cm^2^. The lattice and electrons reach full thermal equilibrium within a few picoseconds in low-fluence case, while the duration is much longer (100 ps) for the strong-pump case [Fig. S7 (a)]. The lattice temperature gradually decreases from the surface into the material. The impact of such a temperature gradient on the actual thermal emitted power is discussed in Sec. S6.

Figure S6 shows the calculated $n$, $T_{e},$ and $T_{l}$ for GaAs with a pump fluence of 0.028 (top) and 0.21 mJ/cm^2^ (bottom). Two major differences between GaAs and Si are worth noting. First, the linear absorption coefficient is larger for GaAs, leading to a smaller penetration depth. Therefore, the generated free carriers are confined in a narrower region near the surface for GaAs [Fig. S6(a, d)]. Second, GaAs has a larger bandgap than Si. Therefore, the peak hot-carrier temperature of GaAs [~4000 K, Fig. S6(b, e)] is lower than that for Si.


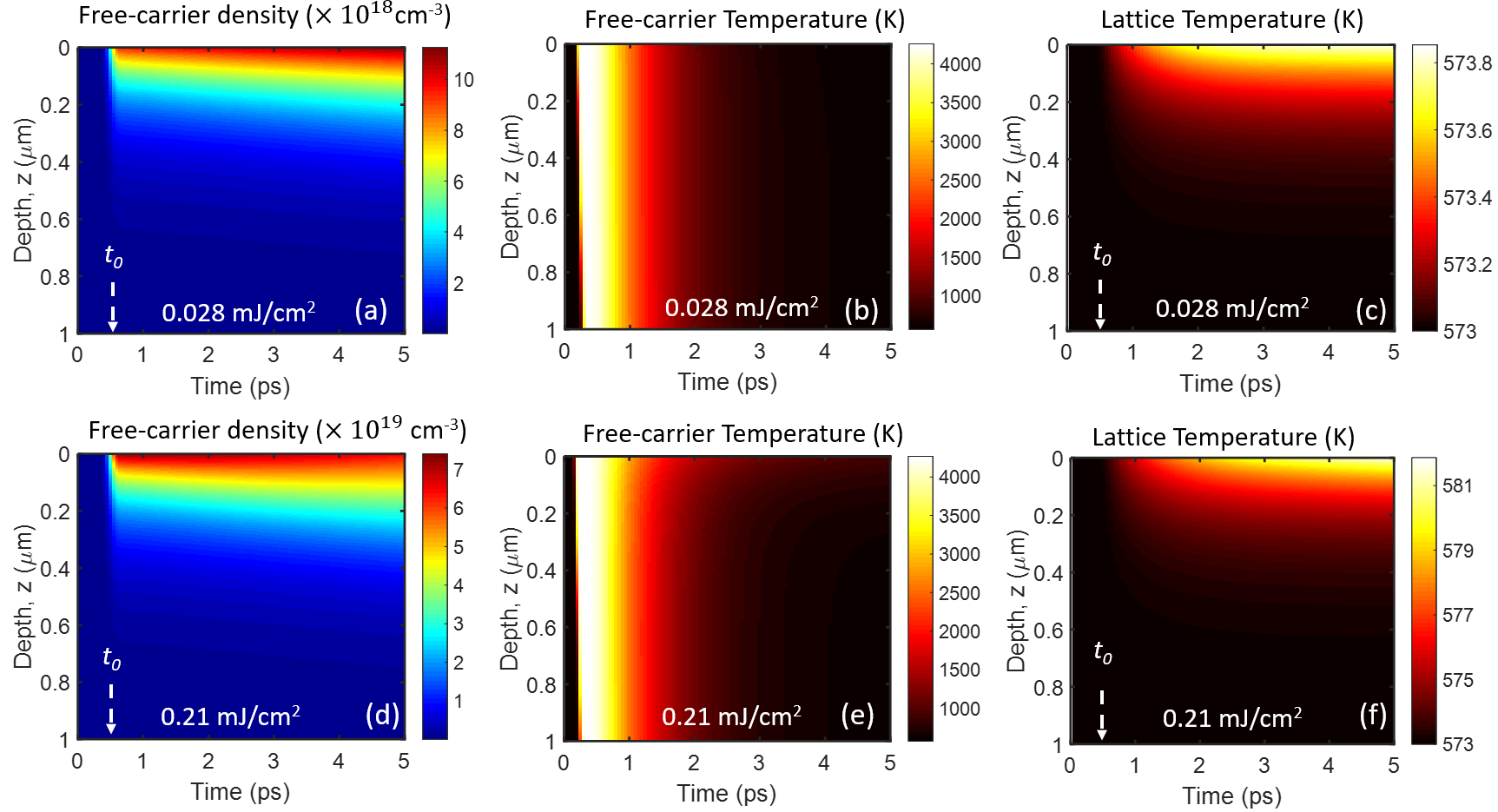


Figure S6. Calculated time- and depth-dependent free-carrier density (a and d), free-carrier temperature (b and e), and lattice temperature (c and f) for a 300 °C GaAs wafer excited by a 200-fs Gaussian pump at 515 nm with fluence of 0.028 (top) and 0.21 mJ/cm^2^ (bottom).


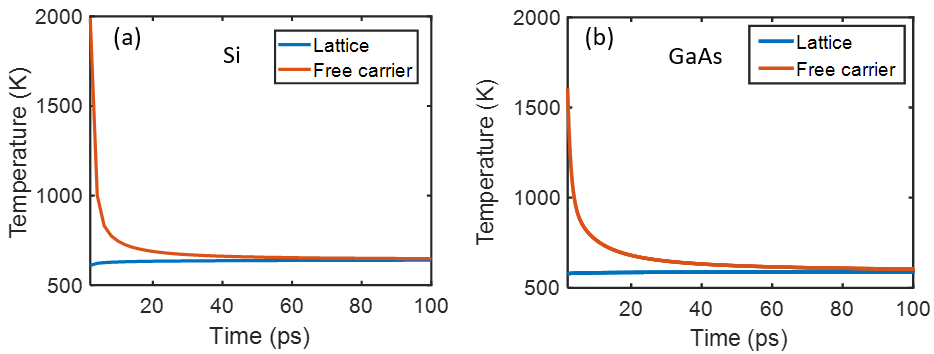


Figure S7. (a) Calculated lattice (blue) and free-carrier (red) temperature at the surface ($z=$ 0 µm) for a 300 °C Si wafer excited by a 200-fs Gaussian pump at a central wavelength of 515 nm with fluence of 1.92 mJ/cm^2^. (b) Same as (a), but with a GaAs wafer with pump fluence of 0.21 mJ/cm^2^.

Figure S8(a) shows the calculated free-carrier density for Si [Fig. S5 (d)] after the pump pulse, from 0.25 to 30 ns. Shortly after the pump, the free carriers are confined within a < 1-$\mu$m-thick layer. The highly confined free carriers quickly diffuse within a few nanoseconds, causing the carrier density to significantly decrease. After 20 ns, the change of carrier density over time becomes much smaller. This is partially due to the relative weaker effect of diffusion, but mainly due to the slow free-carrier recombination of Si (carrier lifetime of a few $\mu$s). Figure S8(b) shows the case for GaAs [Fig. S6 (d)]. The carrier dynamics for GaAs within the first 5 ns are similar to that of Si. However, due to the fast carrier recombination for GaAs (lifetime of a few nanoseconds), the carrier density keeps decreasing even when the effect of carrier diffusion becomes very small (at $t>$ 10 ns).

**
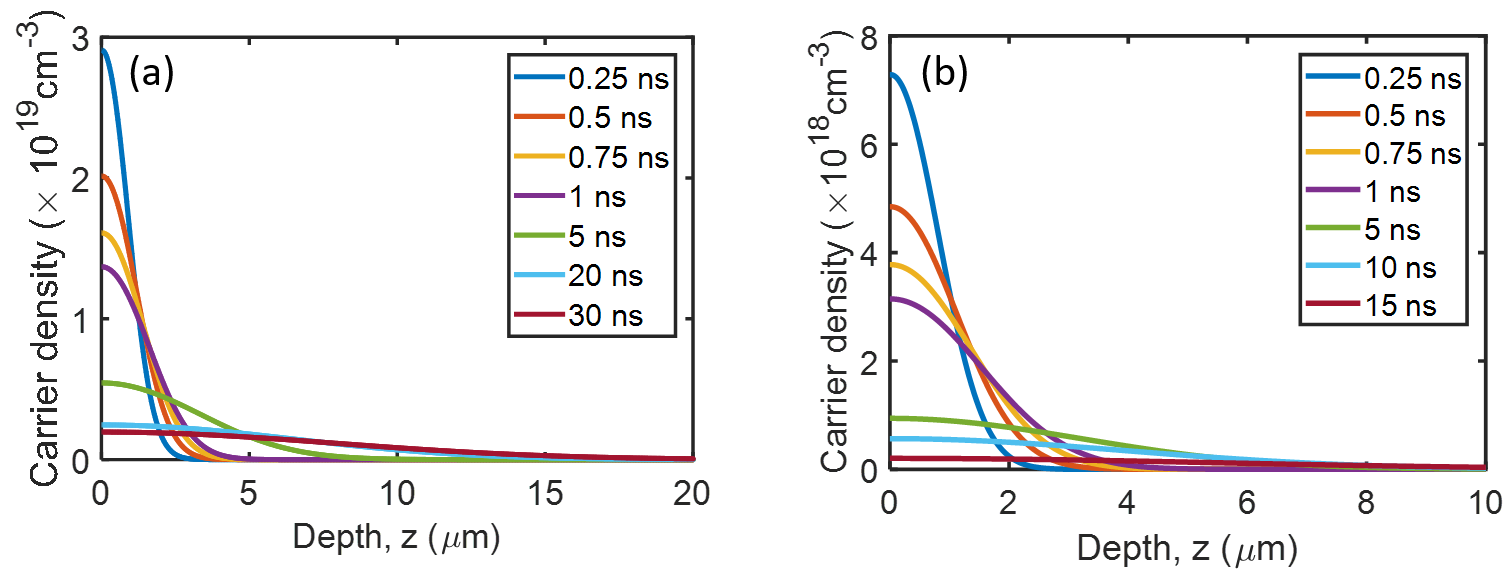
**

Figure S8. Calculated free-carrier density distribution at different times after the pump for (a) a Si wafer with pump fluence of 1.92 mJ/cm^2^, and (b) a GaAs wafer with pump fluence of 0.21 mJ/cm^2^.

Once the free-carrier temperature and the material properties are known, the corresponding thermal emission can be calculated. Here we show the integrated thermal-emission power within the spectral range from 3 to 11 μm (matching our detector wavelength range) for a 300 °C GaAs wafer with pump fluence of 0.21 mJ/cm^2^. The emitting area is 14 mm^2^ and the solid angle over which we integrate is 0.2 sr. Figure S9 shows the calculated thermal emission from hot carriers, after the carriers have thermalized (after100 ps), and the total power that should be detected by our experimental setup. The total detected power in Fig. S9(c) is obtained by convolving (a) and (b) with the temporal response function of our detector (Boston Electronics PVI-4TE-10.6, 0.5 mm $\times$ 0.5 mm), which is a Gaussian function with a width of 1 ns.


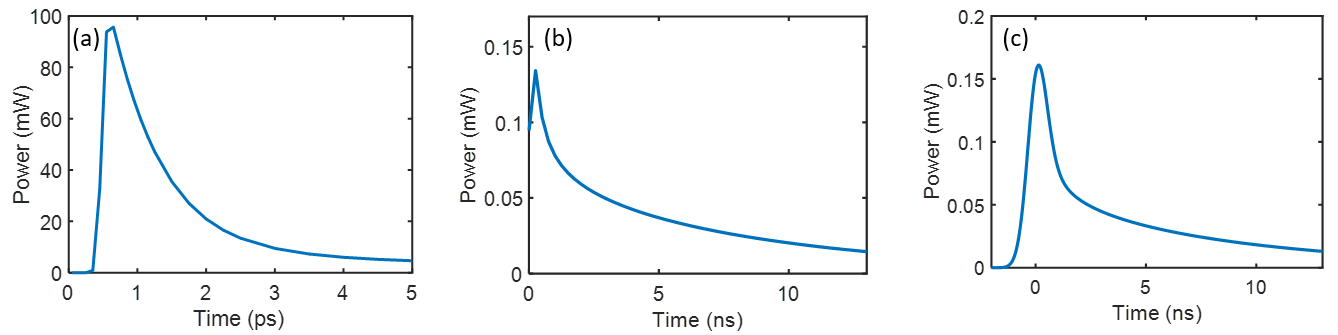


Figure S9. Calculated integrated pulsed thermal emission within the spectral range from 3 to 11 μm (matching our detector bandwidth) for a 300 °C GaAs wafer with pump fluence of 0.21 mJ/cm^2^. (a) and (b) show the emission from hot carriers and after the carriers have thermalized (the plot starts at time = 100 ps), respectively. The emitting area is 14 mm^2^ and we integrate over a solid angle of 0.2 sr. The calculated total signal from the detector is shown in (c).

Note that since the thermal energy from the hot carriers escapes from beneath the flat wafer surface into free space, we expect the emission from hot carriers to be at least somewhat angle- and polarization-dependent. Such dependence is related to the material properties of the wafer. In Fig. S10, we plot the calculated power due to hot-carrier thermal emission for a GaAs wafer at 300 ºC with pump fluence of 0.21 mJ/cm^2^. The calculation was for an emitting area of 14 mm^2^ into a solid angle of 0.2 sr for different angles and polarizations. For angles smaller than ~45º, the angle- and polarization dependence is quite small.

Figure S10. Calculated integrated pulsed thermal emission from hot carriers for different angles and polarizations within the spectral range from 3 to 11 μm (matching our detector bandwidth) for a 300 °C GaAs wafer with pump fluence of 0.21 mJ/cm^2^. The emitting area is 14 mm^2^ and we integrate over a solid angle of 0.2 sr.

**S5: Estimation of the conductive cooling rate of the lattice**

As shown in the simulation [Figs. S5(c) and S6(c)], the heating of the lattice due to optical pumping is negligible when the pump fluence is low. However, there can be non-negligible laser-induced heating of the lattice when the pump power is sufficiently high. Indeed, our model predicts increases of the lattice temperature by 50 K for Si with pump fluence of 1.92 mJ/cm^2^ [Fig. S5 (f)], and by 9 K for GaAs with pump fluence of 0.21 mJ/cm^2^ [Fig. S6 (f)].

To verify that the nanosecond modulation of thermal emission measured comes from the emissivity change due to free-carrier dynamics and not from temperature changes of the lattice, we estimated the cooling rate of the heated wafer by performing heat-transfer simulations. Figure S11 shows the schematic of the structure with some initial temperature distribution due to the pump. We model a thin layer of material representing the laser-heated portion ($\Delta z \sim$0.5 for GaAs and 1 $\mu$m for Si, according to the calculated results from Fig. S5 and S6) on top of a very thick layer of material representing the remaining wafer.


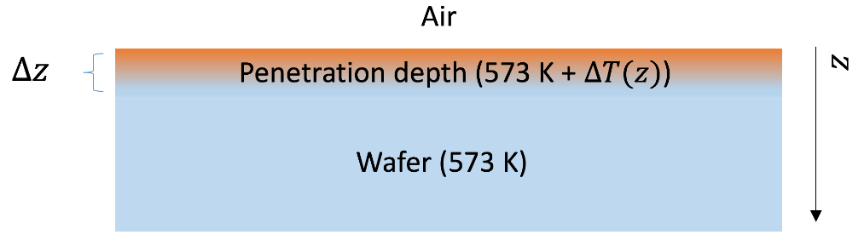


Figure S11. Initial temperature distribution after pump-laser illumination, for heat-transfer simulations.

The lattice temperature of the structure was calculated using the one-dimensional heat equation:

$\frac{\partial T(t,z)}{\partial t}=\frac{k}{\rho C_{p}}\frac{\partial^{2}T(t,z)}{\partial z^{2}}$ (S10)

where $k, \rho,$ and $C_{p}$represent the thermal conductivity, density, and heat capacity of the material, respectively. For the structure shown in Fig. S11, two cooling channels exist: (1) conductive cooling into the substrate and (2) convective cooling into the surrounding air. The thermal conductivities and heat capacity for Si and GaAs we used are in Table S1, while the density are shown in Table S3 (taken from Appendix G of Ref. [11]). The initial temperature distribution was chosen according to the lattice temperature at the end of the FDTD simulation at 100 ps for Si and GaAs.

Table S3: Heat-transfer parameters

| Properties | Silicon | Gallium Arsenide |
| --- | --- | --- |
| $\rho$(kg/m^3^) | 2330 | 5317 |

Equation S11 was solved for a $L=10$µm thick wafer, with a convective heat-transfer boundary condition at $z=0$µm and a fixed temperature $T=573 K$ at $z=10$µm, which corresponds to the temperature of the stage. Figure S12 (a, b) shows the calculated temperature distribution of Si and GaAs for the first 30 and 15 ns, respectively. As shown here, the changes in the lattice temperature over this time scale can be neglected.


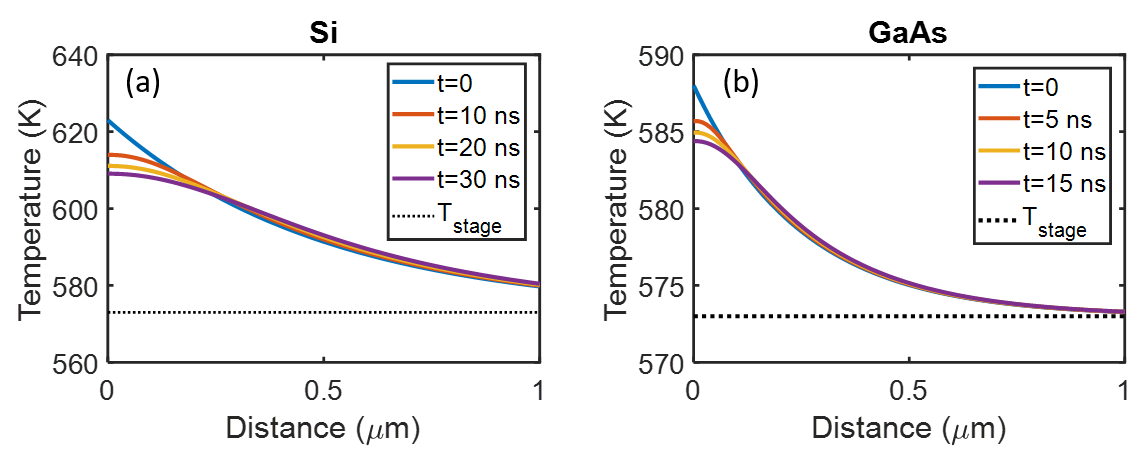


Figure S12. Calculated temperature of the wafer as a function of the distance from the surface for (a) Si with a pump fluence of 1.92 mJ/cm^2^ and (b) GaAs with a pump fluence of 0.21 mJ/cm^2^.

Further evidence that lattice temperature change does not play a significant role comes from the comparison in the experiment data between Si and GaAs in Figs. 1 and S1. GaAs and Si have similar thermal properties, and therefore similar cooling dynamics, but the decay rate of the thermal-emission signal in these experiments is very different between these two materials. Therefore, the nanosecond-scale modulation of thermal emission is clearly a result of the change in emissivity, rather than the temperature.

Figure S13 shows the calculated temperature distributions in the Si and GaAs wafers after a significantly longer period of time. As shown here, the entire substrate cools down after about 100 µs for Si and 200 µs for GaAs. The laser repetition rate is 1 kHz in our experiment, corresponding to a separation time between adjacent pulses of 1 ms. Therefore, the accumulated heating effect due to repetitive pulses can be neglected.


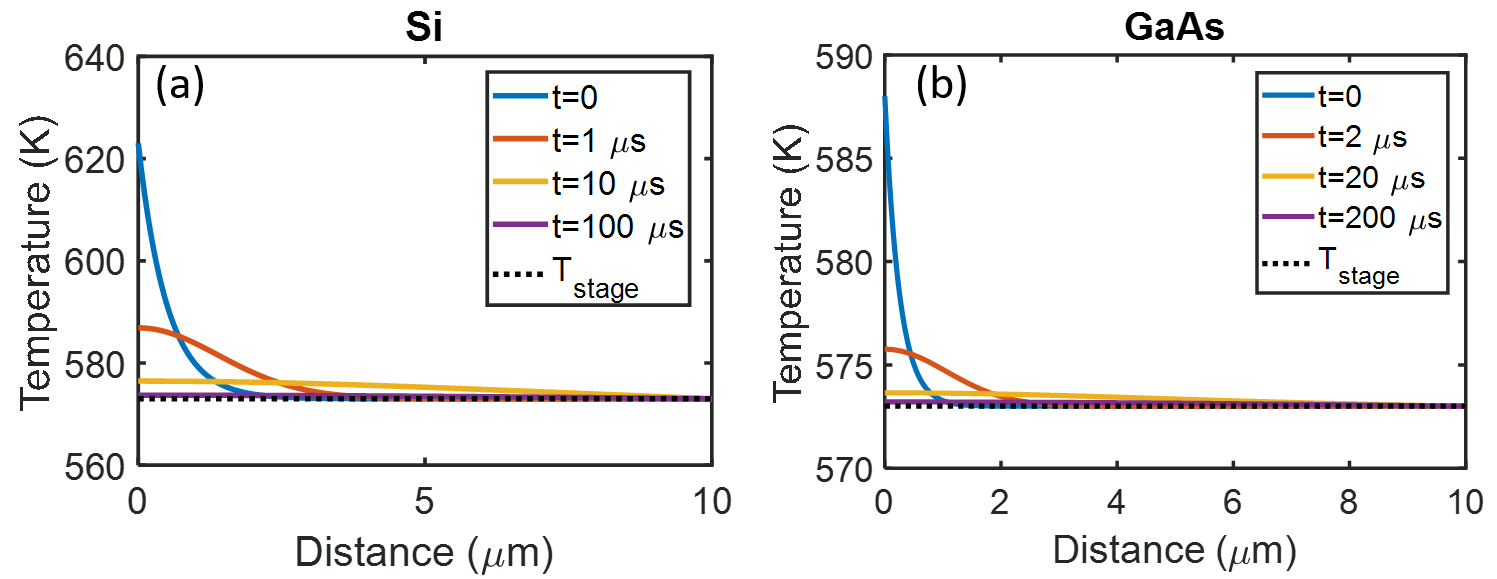


Figure S13. Calculated lattice temperature as a function of wafer depth after a longer period has elapsed post pumping for (a) Si and (b) GaAs.

**S6: Thermal emission from objects with depth-dependent temperature distributions**

The intensity of the pump laser is attenuated as it propagates into the material. Due to the gradient pump intensity, the heating of the free carriers and the lattice is not uniform [Figs. S5(f) and S6(f)]. For a system with a non-uniform temperature distribution, the thermally emitted power cannot be trivially calculated via Kirchhoff’s Law, *i.e.*, by multiplying the blackbody radiation distribution at a particular temperature by the emissivity. The situation is even more complicated immediately after the pump, when the free carriers at a much higher temperature than the lattice. Instead, we calculate the thermal emission directly, using the method described in Section S3, to obtain the emitted power from such a system. Note that here the optical properties are also depth-dependent.

Figure S14 compares the theoretical thermally emitted power from a GaAs wafer long after the pump is extinguished ($t>0.1$ ns, so no hot carriers), calculated both directly and using Kirchhoff’s law, for pump fluences of 0.028, 0.21 and 2.1 mJ/cm^2^ to mimic our experimental conditions. In these calculations, an emitting solid angle of 0.2 sr and an emitting area of 14 mm^2^ is assumed. In the Kirchhoff’s-law calculation, the temperature of the surface ($z=0 \mu$m) at $t_{0}=$100 ps (when free carriers are in thermal equilibrium with the lattice) was used, while in the direct calculation, the actual lattice temperature at each depth was used. The lattice temperatures for the pump fluence 0.028 and 0.21 mJ/cm^2^ are shown in Fig. S6(c, f), respectively, while the lattice temperature for the highest pump fluence of 2.1 mJ/cm^2^ is shown in Fig. S14(d).

As shown in Fig. S14(a), the indirect and direct ways of calculating thermally emitted power are identical for the lowest pump fluence. This is expected since the gradient in the lattice temperature is minimal with a total temperature difference of less than 1 K$,$or less than 0.2 % of the relative temperature change [Fig. S6(c)]. The effect of the gradient temperature distribution on the emitted power becomes noticeable as the pump fluence is increased to 0.21 mJ/cm^2^, and the temperature difference between the surface and substrate is ~ 10 K. As a result, the emitted power from the direct calculation is about 5% smaller than that from Kirchhoff’s law. This effect becomes very significant for the highest pump fluence of 2.1 mJ/cm^2^, where the temperature drop is more than 100 K. In this case, the directly calculated emitted power is about 50% of the value calculated indirectly via Kirchhoff’s law.


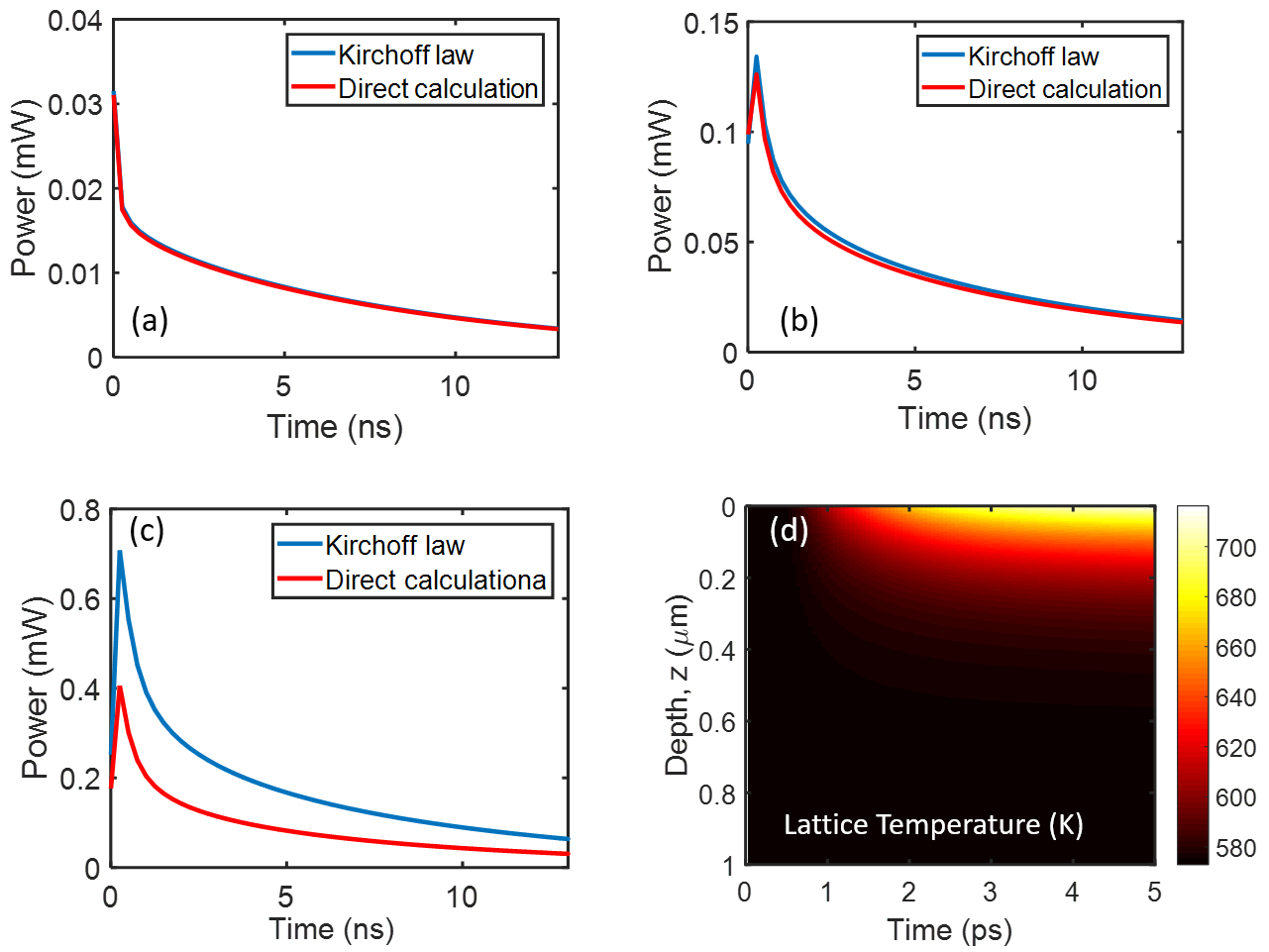


Figure S14. Calculated thermally emitted power from a GaAs wafer long after the pump ($t>0.25$ ns), using Kirchhoff’s law (blue curve, using the surface lattice temperature) and direct calculation (red) with pump fluence of 0.028, 0.21 and 2.1 mJ/cm^2^, respectively. (d) The lattice temperature for a 300 °C GaAs wafer excited by a 200 fs Gaussian pump at 515 nm with a fluence of 2.1 mJ/cm^2^.

**S7: Conversion efficiency from the visible pump pulse to infrared thermal pulse**

One can view this process of generating mid-infrared pulses from ones in the visible as an unconventional type of frequency conversion. One potential way to quantify the conversion efficiency is to take the ratio of the integrated power of the thermal pulse (i.e., the pulse energy) over that of the pump pulse. The conversion efficiency of this process depends on a lot of factors, such as the pump power and wavelength, the material system used, and the sample temperature. For example, in our experiments, the conversion efficiency for Si emitters is much higher than for GaAs emitters due to the longer and stronger mid-IR pulse from Si. The conversion efficiency for emitters at 300 ºC sample is higher than for the same emitters at 200 ºC because the thermal emission is determined by the stage temperature. Also, two distinct pulses are created in our experiments: one from hot carriers within the dielectric environment resulting from the presence of the carriers and the lattice, and one from the semiconductor in thermal equilibrium (i.e., the temperature of the carriers and the lattice is equal). Here, we refer to the former as *hot-carrier thermal emission*, and the latter as *thermal-equilibrium thermal emission*.

As an example, we consider the case of 300 ºC GaAs with pump fluence of 0.21 mJ/cm^2^. Figure S9 shows the calculated thermal-emission power from an emitting area of 14 mm^2^ into a solid angle of 0.2 sr in the spectral range of 3-11 micron (the bandwidth of our detector). For thermal-equilibrium thermal emission (which results in the vast majority of the power in the nanosecond mid-IR pulse), most of the energy is contained in this spectral range. Therefore, Fig. S9(b) represents the total power in the mid-IR pulse. For hot-carrier thermal emission, more energy is in the near-IR range due to their high temperature. Therefore, Fig. S9(a) only represents a small portion of the total power of the picosecond hot-carrier pulse. We plot the calculated total emission power of the hot carriers in the following figure together with the integrated emission power in the spectral range of 3-11 micron. Indeed, the total power is about 6 times higher than that in the 3-11 micron spectral range.

Figure S15. Calculated hot-carrier thermal-emission pulse from a 300 °C GaAs wafer with pump fluence of 0.21 mJ/cm^2^. The emitting area is 14 mm^2^ and we integrate over a solid angle of 0.2 sr. The integrated emission power in the spectral range of 3-11 micron is shown by the black dotted curve.

The total energy contained in the picosecond hot-carrier thermal-emission pulse is about 0.4 pJ. The total energy in the nanosecond thermal-equilibrium thermal-emission pulse (Fig. S9(b)) is about 0.5 pJ. The total energy in the pump pulse at 515 nm in this case is about 0.05 mJ. Therefore the conversion efficiency for GaAs here is about 0.9 pJ/0.05 mJ ~ 2 $\times{10}^{-8}$. In this case, the fraction of energy into the hot-carrier picosecond hot-carrier thermal-emission pulse and the nanosecond thermal-equilibrium thermal-pulse are roughly equal.

We can do similar analysis for Si. Because Si has a much longer free-carrier lifetime than GaAs, one has to measure a much wider temporal window to properly account for the total pulse energy. In the following figure, we plot the experimentally calibrated thermal-emission power from Si from an emitting area of 1.4 mm^2^ into a solid angle of 0.2 sr, at stage temperatures of 200 ºC with a pump fluence of 2.14 mJ/cm^2^ (extended version of Fig. S1 (c)).

Figure S16. Experimentally calibrated thermal-emission power from Si from an emitting area of 1.4 mm^2^ into a solid angle of 0.2 sr, at a stage temperature of 200 ºC, with a pump fluence of 2.14 mJ/cm^2^ (extended version of Fig. S1 (c)).

As shown here, due to the long free-carrier lifetime, the thermal pulse (from thermal-equilibrium thermal emission) from Si lasts for more than 50 microseconds. The total energy contained in this thermal pulse is about 0.3 nJ. The total energy within the picosecond hot-carrier thermal-emission pulse is much smaller than that of the thermal-equilibrium thermal-emission pulse. The energy in our 515-nm pump pulse in this case is about 0.05 mJ. Therefore, the conversion efficiency for Si in this case is about 0.3 nJ/0.05 mJ ~ 6 $\times{10}^{-6}$.

Note that the efficiencies estimated above only consider the portion of the thermal-emission pulse radiated into a solid angle of 0.2 sr. The actual conversion efficiencies (i.e., over a solid angle of 2$\pi$ sr) would be approximately 6 $\times{10}^{-7}$and 2 $\times{10}^{-5}$ for GaAs and Si, respectively. Indeed, the conversion efficiency we demonstrated here is not very high, but we can consider a number of approaches that may be used in future extensions of this approach to increase the conversion efficiency:

#1：The power of the thermal-equilibrium thermal emission pulse (and therefore the conversion efficiency) can be increased by increasing the sample temperature. The rate of increase can be approximated using the Stefan-Boltzmann law, and roughly scales as T^4^. As demonstrated in this work, the power of the thermal-equilibrium thermal-emission pulse increases by a factor of 2 when the sample temperature is increased from 200 to 300 ºC, agreeing with the T^4^ prediction: (300+273)^4^/(200+273)^4^ ~ 2. If one could run the same experiment at a much higher temperature, a significant increase of efficiency can be expected. For example, if heating the sample to 1000 ºC, the power of the mid-IR pulse would be increased by roughly 50 times compared to the 200 ºC case. Even higher temperatures may be possible, though they likely would require the use of an inert-gas chamber (like for an incandescent light bulb) and refractory materials.

#2：Another way to increase the conversion efficiency is to decrease the pump-power requirement to induce the change of emissivity. It may be difficult to accomplish very large changes in emissivity with smaller pump powers using conventional semiconductors, but this can be accomplished using ,e.g., materials with phase transitions, biased close to their transition. One example of such a material that we studied extensively in the past is vanadium dioxide (VO_2_), described e.g., in ref. [S30] and [S31]. When biasing the sample temperature close to the temperature of the transition, the transition (and therefore change in emissivity) can occur with a weak pump [S32]. Therefore, similar behavior to what is demonstrated in our present paper is expected to occur for much weaker pump power, increasing the efficiency dramatically.

We note that VO_2_ in particular is likely not a good candidate material for this particular demonstration, because the required bias temperature must be just below its transition temperature of ~70 ºC. Therefore, the thermal power from a VO_2_ emitter would be quite low. However, materials such as NbO_2_ feature phase transitions at much higher temperatures (~900 ºC, from [S33]), and such materials are likely much better candidates.

#3：There are also possible ways to increase the total energy of the hot-carrier thermal emission pulse. The total energy of the picosecond hot-carrier thermal emission pulse depends both on the temperature and lifetime of the hot carriers. Therefore, either increasing the temperature or the lifetime can increase the total pulse energy. The hot-carrier temperature can be increased by choosing a material with a smaller bandgap since $T \sim(hf-E_{g})/3k_{B}$. The hot-carrier lifetime can be increased by choosing a material with a smaller election-phonon scattering rate (such as ~100 picoseconds hot-carrier lifetime observed in perovskites [S34]) or decreasing the electron-phonon scattering rate via cooling the material to low temperatures.

##### **Supplementary references**

[S1] J. K. Chen, D. Y. Tzou, and J. E. Beraun, “Numerical investigation of ultrashort laser damage in semiconductors,” *Int. J. Heat Mass Transf.*, vol. 48, no. 3–4, pp. 501–509, 2005.

[S2] J. S. Blakemore, “Semiconducting and other major properties of gallium arsenide,” *J. Appl. Phys.*, vol. 53, no. 10, pp. R123–R181, Oct. 1982.

[S3] V. M. Glazov and A. S. Pashinkin, “Thermal expansion and heat capacity of GaAs and InAs,” *Inorg. Mater.*, vol. 36, no. 3, pp. 225–231, Mar. 2000.

[S4] J. R. Goldman and J. A. Prybyla, “Ultrafast hot-electron dynamics in silicon,” *Semicond. Sci. Technol.*, vol. 9, no. 5S, pp. 694–696, May 1994.

[S5] T. Sjodin, H. Petek, and H.-L. Dai, “Ultrafast Carrier Dynamics in Silicon: A Two-Color Transient Reflection Grating Study on a ( 111 ) Surface,” *Phys. Rev. Lett.*, vol. 81, no. 25, pp. 5664–5667, Dec. 1998.

[S6] M. Bernardi, D. Vigil-Fowler, J. Lischner, J. B. Neaton, and S. G. Louie, “*Ab Initio* Study of Hot Carriers in the First Picosecond after Sunlight Absorption in Silicon,” *Phys. Rev. Lett.*, vol. 112, no. 25, p. 257402, Jun. 2014.

[S7] W.-Z. Lin, R. W. Schoenlein, J. G. Fujimoto, and E. P. Ippen, “Femtosecond absorption saturation studies of hot carriers in GaAs and AlGaAs,” *IEEE J. Quantum Electron.*, vol. 24, no. 2, pp. 267–275, Feb. 1988.

[S8] U. Hohenester, P. Supancic, P. Kocevar, X. Q. Zhou, W. Kütt, and H. Kurz, “Subpicosecond thermalization and relaxation of highly photoexcited electrons and holes in intrinsic and *p* -type GaAs and InP,” *Phys. Rev. B*, vol. 47, no. 20, pp. 13233–13245, May 1993.

[S9] R. A. Sinton and R. M. Swanson, “Recombination in highly injected silicon,” *IEEE Trans. Electron Devices*, vol. 34, no. 6, pp. 1380–1389, Jun. 1987.

[S10] D. Steiauf, E. Kioupakis, and C. G. Van de Walle, “Auger Recombination in GaAs from First Principles,” *ACS Photonics*, vol. 1, no. 8, pp. 643–646, Aug. 2014.

[S11] S. M. Sze and K. K. Ng, *Physics of Semiconductor Devices*. Hoboken, NJ, USA: John Wiley & Sons, Inc., 2006.

[S12] M. Stobbe, R. Redmer, and W. Schattke, “Impact ionization rate in GaAs,” *Phys. Rev. B*, vol. 49, no. 7, pp. 4494–4500, Feb. 1994.

[S13] B. A. Ruzicka, L. K. Werake, H. Samassekou, and H. Zhao, “Ambipolar diffusion of photoexcited carriers in bulk GaAs,” *Appl. Phys. Lett.*, vol. 97, no. 26, p. 262119, Dec. 2010.

[S14] Y. P. Varshni, “Temperature dependence of the energy gap in semiconductors,” *Physica*, vol. 34, no. 1, pp. 149–154, Jan. 1967.

[S15] S. Tiwari and S. L. Wright, “Material properties of *p* ‐type GaAs at large dopings,” *Appl. Phys. Lett.*, vol. 56, no. 6, pp. 563–565, Feb. 1990.

[S16] J. Šik, J. Hora, and J. Humlı́ček, “Optical functions of silicon at high temperatures,” *J. Appl. Phys.*, vol. 84, no. 11, p. 6291, Nov. 1998.

[S17] G. E. Jellison, “Optical functions of GaAs, GaP, and Ge determined by two-channel polarization modulation ellipsometry,” *Opt. Mater. (Amst).*, vol. 1, no. 3, pp. 151–160, Sep. 1992.

[S18] B. S. Wherrett, “Scaling rules for multiphoton interband absorption in semiconductors,” *J. Opt. Soc. Am. B*, vol. 1, no. 1, p. 67, Mar. 1984.

[S19] S. Krishnamurthy, Z. G. Yu, L. P. Gonzalez, and S. Guha, “Temperature- and wavelength-dependent two-photon and free-carrier absorption in GaAs, InP, GaInAs, and InAsP,” *J. Appl. Phys.*, vol. 109, no. 3, p. 033102, Feb. 2011.

[S20] G. C. Valley, T. F. Boggess, J. Dubard, and A. L. Smirl, “Picosecond pump‐probe technique to measure deep‐level, free‐carrier, and two photon cross sections in GaAs,” *J. Appl. Phys.*, vol. 66, no. 6, pp. 2407–2413, Sep. 1989.

[S21] D. M. Caughey and R. E. Thomas, “Carrier mobilities in silicon empirically related to doping and field,” *Proc. IEEE*, vol. 55, no. 12, pp. 2192–2193, 1967.

[S22] M. C. Nuss, D. H. Auston, and F. Capasso, “Direct Subpicosecond Measurement of Carrier Mobility of Photoexcited Electrons in Gallium Arsenide,” *Phys. Rev. Lett.*, vol. 58, no. 22, pp. 2355–2358, Jun. 1987.

[S23] M. Bernardi, D. Vigil-Fowler, C. S. Ong, J. B. Neaton, and S. G. Louie, “Ab initio study of hot electrons in GaAs.,” *Proc. Natl. Acad. Sci. U. S. A.*, vol. 112, no. 17, pp. 5291–6, Apr. 2015.

[S24] Z. Mics, A. D’Angio, S. A. Jensen, M. Bonn, and D. Turchinovich, “Density-dependent electron scattering in photoexcited GaAs in strongly diffusive regime,” *Appl. Phys. Lett.*, vol. 102, no. 23, p. 231120, Jun. 2013.

[S25] F. Meng, M. D. Thomson, B. E. Sernelius, M. Jörger, and H. G. Roskos, “Ultrafast dynamic conductivity and scattering rate saturation of photoexcited charge carriers in silicon investigated with a midinfrared continuum probe,” *Phys. Rev. B*, vol. 91, no. 7, p. 075201, Feb. 2015.

[S26] M. Francoeur, M. Pinar Mengüç, and R. Vaillon, “Solution of near-field thermal radiation in one-dimensional layered media using dyadic Green’s functions and the scattering matrix method,” *J. Quant. Spectrosc. Radiat. Transf.*, vol. 110, no. 18, pp. 2002–2018, 2009.

[S27] L. Novotny and B. Hecht, *Principles of nano-optics*. Cambridge, 2012.

[S28] C. Balanis, *Advanced engineering electromagnetics*. John Wiley & Sons, 1999.

[S29] M. Achermann, A. P. Bartko, J. A. Hollingsworth, and V. I. Klimov, “The effect of Auger heating on intraband carrier relaxation in semiconductor quantum rods,” *Nat. Phys.*, vol. 2, no. 8, pp. 557–561, Aug. 2006.

[S30] Kats, Mikhail A., et al. "Vanadium dioxide as a natural disordered metamaterial: perfect thermal emission and large broadband negative differential thermal emittance." *Physical Review X* 3.4 (2013): 041004

[S31] Wan, Chenghao, et al. "Optical properties of thin-film vanadium dioxide from the visible to the far infrared." *arXiv preprint arXiv:1901.02517* (2019).

[S32] Cocker, T. L., et al. "Phase diagram of the ultrafast photoinduced insulator-metal transition in vanadium dioxide." *Physical Review B* 85.15 (2012): 155120.

[S33] Sakata, T., K. Sakata, and I. Nishida. "Study of phase transition in NbO2." *physica status solidi (b)* 20.2 (1967): K155-K157.

[S34] Yang, Ye, et al. "Observation of a hot-phonon bottleneck in lead-iodide perovskites." *Nature Photonics* 10.1 (2016): 53-59.
